# Loss of LAMP5 interneurons drives neuronal network dysfunction in Alzheimer’s disease

**DOI:** 10.1007/s00401-022-02457-w

**Published:** 2022-07-03

**Authors:** Yuanyuan Deng, Mian Bi, Fabien Delerue, Shelley L. Forrest, Gabriella Chan, Julia van der Hoven, Annika van Hummel, Astrid F. Feiten, Seojin Lee, Ivan Martinez-Valbuena, Tim Karl, Gabor G. Kovacs, Grant Morahan, Yazi D. Ke, Lars M. Ittner

**Affiliations:** 1 Dementia Research Centre, Macquarie Medical School, Faculty of Medicine, Health and Human Sciences, Macquarie University, Sydney, NSW 2109 Australia; 2 Tanz Centre for Research in Neurodegenerative Disease, University of Toronto, Toronto, ON M5S 1A1 Canada; 3 School of Medicine, Western Sydney University, Sydney, NSW 2560 Australia; 4 Department of Laboratory Medicine and Pathobiology and Department of Medicine, University of Toronto, Toronto, ON M5S 1A8 Canada; 5 Laboratory Medicine Program and Krembil Brain Institute, University Health Network, Toronto, ON M5S 2S1 Canada; 6 Centre for Diabetes Research, Harry Perkins Institute of Medical Research, Perth, WA 6150 Australia

**Keywords:** Alzheimer’s disease, Amyloid-β, Tau, Hyperexcitation, Neuronal network, LAMP5

## Abstract

In Alzheimer’s disease (AD), where amyloid-β (Aβ) and tau deposits in the brain, hyperexcitation of neuronal networks is an underlying disease mechanism, but its cause remains unclear. Here, we used the Collaborative Cross (CC) forward genetics mouse platform to identify modifier genes of neuronal hyperexcitation. We found LAMP5 as a novel regulator of hyperexcitation in mice, critical for the survival of distinct interneuron populations. Interestingly, synaptic LAMP5 was lost in AD brains and LAMP5 interneurons degenerated in different AD mouse models. Genetic reduction of LAMP5 augmented functional deficits and neuronal network hypersynchronicity in both Aβ- and tau-driven AD mouse models. To this end, our work defines the first specific function of LAMP5 interneurons in neuronal network hyperexcitation in AD and dementia with tau pathology.

**Supplementary Information:**

The online version contains supplementary material available at 10.1007/s00401-022-02457-w.

## Introduction

Alzheimer’s disease (AD) is the most common neurological disease and an increasing global health problem [7]. Cognitive decline in AD is linked to synaptic and neuronal loss as well as the deposition of Αβ in extracellular plaques and the microtubule-associated protein tau in intraneuronal fibrillar tangles [16, 27, 34]. While Aβ is derived from the transmembrane Aβ-precursor protein (APP) by proteolytic cleavage and accumulates in AD brains due to increased formation and/or reduced clearance [24], tau undergoes aberrant phosphorylation that interferes with its ability to maintain its physiological functions and makes it prone to aggregation forming toxic species [16]. With current therapeutic options being of limited efficacy, therapies targeting neuronal network dysfunction has gained traction [39], though molecular/cellular targets remain poorly defined.

Others and we have previously reported tau-dependent neuronal hyperexcitation as a pathomechanism contributing to functional deficits in Aβ-induced AD mouse models [16, 18, 29, 32]. Furthermore, neuronal hyperexcitation contributes to deficits in tau-dependent dementia models [9, 25, 30]. This includes neuronal network dysfunction with spontaneous non-convulsive seizures, that have been reported in both AD patients and mouse models [3, 17, 46]. However, the cellular events that mediate neuronal network dysfunction remain unclear.

Here, we used forward genetics in mice to identify modifier genes of neuronal hyperexcitation and reveal novel pathways that contribute to neuronal network dysfunction in AD. The combination of both Aβ and tau-dependent transgenic mouse models of AD with validation in human donor brain tissue led us to identify the loss of distinct interneuronal sub-populations likely contributing to neuronal network failure in AD pathogenesis.

## Materials and methods

### Mice

**Fig. 1:**
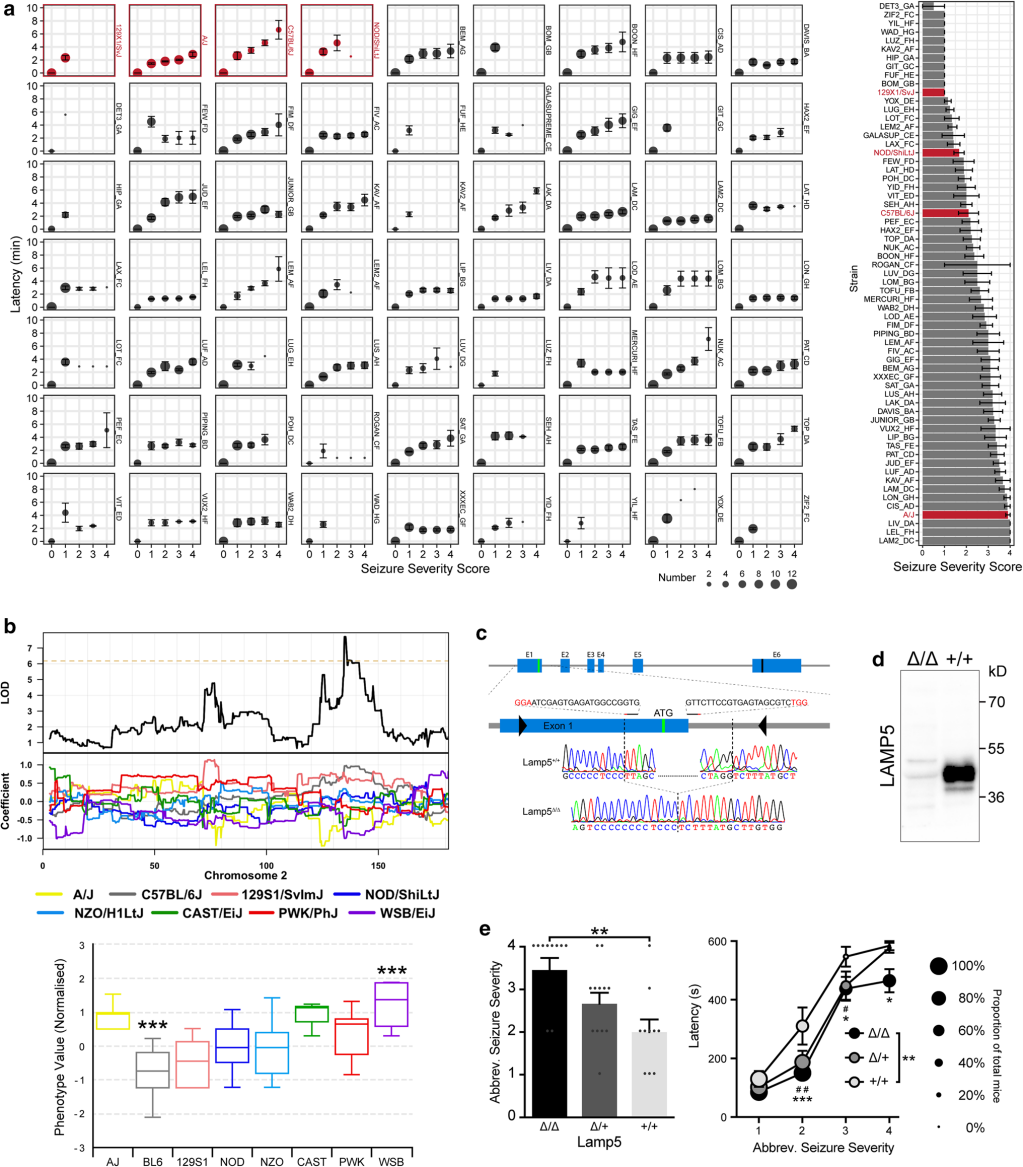
LAMP5 limits neuronal hyperexcitation. **a** Latency to develop more severe seizures (left) and ranking of mean seizure severity score (right) reached within 10 min after intraperitoneal administration of 50 mg/kg pentylenetetrazole (PTZ) in 59 collaborative cross (CC; black) and 4 inbred strains (red). Symbol size indicate number of mice that reached the indicated seizure severity score. **b** QTL mapping using mean seizure severity identified a seizure susceptibility locus on the murine chromosome 2 (top/middle) including contribution of the 8 CC background strains to this locus. **c** Gene targeting approach to generate Lamp5^Δ/Δ^ mice by deleting 250 bp of the exon1/intron1 junction including the translational start codon (ATG) of the murine *Lamp5* locus, as confirmed by genomic sequencing. **d** Western blotting confirms absence of LAMP5 in Lamp5^Δ/Δ^ mice. **e** Mean seizure severity (left) and latency to more severe seizures (right) in response to PTZ in Lamp5^+/+^, Lamp5^Δ/+^, Lamp5^Δ/Δ^ mice (*n* = 10–12; *p < 0.05; **p < 0.01; ***p < 0.001; one-way ANOVA). Latency symbol sizes indicate proportion of mice reaching each seizure score

Animal experiments were approved by the Animal Ethics Committee of Macquarie University. APP23 [36], TAU58 [44], APP/PS1 [2] and *Mapt*-null (tau^–/–^) mice [42] were previously described. The Collaborative Cross (CC) platform has been reported in detail before [6]. CC mice were obtained from the Australian Resources Centre (Perth, Australia) and directly used for experiments. Lamp5^Δ/Δ^ mice were generated using CRISPR/Cas9 genome editing [49]. Briefly, cytoplasmic microinjection of Cas9 mRNA (50 ng/µl) and *Lamp5* gene-specific guide RNAs (sgRNA1 5′-GTGGCCGGTAGAGTGAGCTA-3′ and sgRNA2 5′-GTTCTTCCGTGAGTAGCGTC-3′; 12.5 ng/µl each) into fertilized C57BL/6 zygotes induced a target deletion of 250 bp comprising the transcriptional start in exon 1 of the *Lamp5* locus (Fig. 1c). First-generation (F1) heterozygotes offspring were obtained from a founder with confirmed homozygous deletions around the ATG initiation codon by Sanger sequencing. F1 *Lamp5*^Δ/+^ mice were sibling-mated to generate all three genotypes: homozygous knockouts *Lamp5*^Δ/Δ^, heterozygous knockouts *Lamp5*^Δ/+^ and non-mutant *Lamp5*^+/+^ mice (Supplementary Fig. 1, online resource). All lines were maintained on a C57Bl/6 background and identified by polymerase chain reaction (PCR). Oligonucleotide primers used for genotyping are described in Supplementary Table 1 (online resource). Mice had *ad libitum* access to water and food and were housed on a 12 h day-night cycle.

### Seizures

Mice were intraperitoneally injected with 50 mg/kg pentylenetetrazole (PTZ, Sigma-Aldrich) in phosphate-buffered saline (PBS) to induce seizures [18]. Animals were video monitored for ten minutes and times to reach individual seizure stage was recorded. Abbreviated Seizure Severity Stage: 0, no seizures; 1, immobility; 2, forelimb clonus; 3, generalized clonus; 4, tonic clonic seizures or worse. Then mice were terminated.

### Quantitative trail locus mapping

All data entry was performed on Excel and later processed in R (version 3.2.3) on RStudio (version 0.99.491) platform. The following packages were used for data processing and visualisation: reshape2, ggplot2, sangerseqR, Biostrings, BiocGenerics, parallel, S4Vectors, stats4, IRanges, XVector. Quantitative trait locus (QTL) mapping was performed using a previously published [31] and pre-established web application platform called GeneMiner (http://130.95.9.22/Geniad2/). Different permutations of phenotype data were imputed to screen for meaningful inputs.

### Human samples

Human brain tissue sections were obtained from the New South Wales Brain Bank and the Toronto UHN Neurodegenerative Disease brain collection. Clinical and neuropathological details are described in Supplementary Table 2 (online resource). All cases were recruited with informed consent through regional brain donor programs. The brain donor programs hold ethics approval through the University Health Network (CAPCR-ID: 20-5258 and University of Toronto (Nr. 39459), Research Ethics Board and Human Ethics Committees of the South Eastern and Illawarra Area Health Service, the University of New South Wales and the University of Sydney. All procedures complied with the statement on human experimentation issued by the National Health and Medical Research Council of Australia. All cases included in this study received a routine pathological diagnosis according to neuropathological consensus recommendations. Alzheimer’s disease cases had Braak NFT stage ≥ 5, Thal phase ≥ 4 or CERAD scores ≥ 3 and control cases had Braak NFT stage ≤ 1, Thal phase ≤ 1 or CREAD scores ≤ 1. Co-existing phosphorylated TDP-43 or a-synuclein immunoreactivity was only in a subset of AD cases.

### Histology

Mice were anaesthetized and transcardially perfused with cold PBS at specified ages. Brains were removed and post-fixed in 4% (w/v) paraformaldehyde (PFA) in PBS overnight. Tissue was processed in an Excelsior tissue processor (Thermo Fisher) followed by paraffin embedding. Brains were sectioned at 4 μm. Brain sections from animals and human patients were stained with primary antibody against LAMP5 (Thermo Fisher), eGFP (Abcam), Aβ (6E10, BioLegend), tau phosphorylated at Ser 214 (pS214, Abcam) and at Ser 422 (pS422, Abcam). For fluorescence staining, sections were incubated with Alexa Fluor conjugated secondary antibodies (Thermo) and 4′,6-diamidine-2′-phenylindole dihydrochloride (DAPI) for 1 h at room temperature. For chromogenic staining, biotinylated secondary antibodies and avidin–biotin complexed to horseradish peroxidase (HRP) from a detection kit (Vector) were applied after primary antibodies. Slides were then incubated with 3,3′-diaminobenzidine (DAB, Vector), followed by a haematoxylin counterstain, and mounted with DPX mounting medium. Aβ plaque load in mouse brains was determined using Thioflavin S staining following a standard protocol. Briefly, deparaffinated and rehydrated brain sections were incubated in pre-filtered 1% (w/v) aqueous Thioflavin-S for 8 min, followed by washing in 80% ethanol and water, before being mounted with aqueous mounting media (Fluoromount-G, Southern Biotech). All staining were either imaged using a BX51 bright field/epifluorescence microscope equipped with a DP70 colour camera (Olympus) or Axio Imager Z1 (Zeiss) or scanned on an Axio Scan.Z1 slide scanner (Zeiss) or Huron TissueScope LE120 slide scanner. Using HuronViewer, five randomly selected areas of interest (AOI, 1000 × 1000 pixel size) from the human putamen and globus pallidus from each case were captured at 21× magnification and exported to GIMP (https://www.gimp.org). The total number of LAMP5 pixels in each AOI was analysed following colour deconvolution and threshold adjustment to remove the background. The total number of pixels in each AOI was averaged across the five AOIs and expressed as % pixel area for each case. For human hippocampus, LAMP5 positive projections were counted manually on five randomly captured images at 20× magnification. For human cortex, LAMP5 positive cells were counted automatically using the Fiji ImageJ image processing software (NIH).

### Aβ levels and pathology

Aβ_42_ levels were examined by ELISA as previously described [18].

### Lamp5 promoter reporter

The *pLamp5*-GFP reporter construct was designed by cloning 1753 bp upstream of the transcriptional start of the murine *Lamp5* gene locus that contained the annotated promoter 5′ of an enhanced green fluorescence protein (eGFP) open reading frame in an AAV expression vector. Cloning was done by VectorBuilder (USA). Packaging of AAV vector was performed as described [12]. Titers were determined by quantitative polymerase chain reaction (qPCR). Twenty microliters (1 × 10^11^ vector genomes) of AAV-*pLamp5*-GFP was bilaterally intravenously injected into the brains of cryo-anaesthetised neonatal mice as described [23].

### Behaviour and memory test

Open Field (OF) testing was done as previously described [20]. Briefly, mice were placed individually in an open arena (40 cm × 40 cm) in dimly lit sound-insulated enclosures and recorded for 10 min. Movements were tracked and analysed using the AnyMaze software (Stölting). Spatial learning and memory formation was assessed in the Morris water maze (MWM) as described before [30, 45]. Briefly, the test equipment consisted of a camera, a Perspex platform (10 cm diameter) and a white circular pool (140 cm diameter and 50 cm height) filled with water containing diluted non-irritant white acrylic-based paint dye. The pool was virtually divided into four equal size quadrants, four different visual cues were placed equidistant of the four quadrants. The platform was hidden in the target quadrant (Q1) and submerged 1 cm below the water. The room was low-lit. Testing was done on eight consecutive days, consist of three tests. Day 1–6 was spatial acquisition with each mouse performing four trials started from a different position in the maze. Individual mice were put into the water facing the wall and allowed 60 s to find the hidden platform where they remained for an additional 60 s. The escape latency was recorded as the time to find the platform. If a mouse failed to reach the platform within 60 s, it was guided to the platform. Probe trials were done on day 7 when each mouse was subjected to two rounds of swim trial (30 s each) without the hidden platform in the maze. The platform entries and time spent in each quadrant were recorded. Day 8 concluded the test with a visual cued test for which the platform was placed back inside, with a visible flag on top, and four trials were performed. The latency to reach the platform was recorded and mice with an average time greater than 20 s were excluded. Movements were tracked and analysed using the AnyMaze software (Stölting).

### Electroencephalography

Hippocampal EEG recording was performed in freely moving mice as previously described by us [17]. Briefly, wire EEG electrodes connected to remote telemetric transmitters (DSI) were implanted in the hippocampus (*x* 2.0, *y* − 2.0, *z* − 2.0 from bregma) and a reference electrode was placed above the cerebellum (*x* 0, *y* − 6.0, *z* 0 from bregma) in anaesthetized mice. Electrodes were fixed in place using polyacrylate followed by wound closure. Ten days after surgery, EEGs and activity of animals was recorded using a DSI wireless receiver setup (DSI) with amplifier matrices using the Dataquest A.R.T. recording software at 500 Hz sampling rate [48]. EEG recording was stopped after 48 h. Animals were perfused with ice-cold PBS and brains extracted for biochemical and histological analysis as stated above. Correct implantation of electrodes was confirmed by haematoxylin–eosin staining of serial sections of paraffin-embedded brains. Only recordings from mice with correct placement of electrodes were used for further analysis.

EEG recordings were analysed using the NeuroScore software v3.2.1 (DSI) with an integrated spike detection module. The number of spikes, spike train duration and number of spikes per train were obtained. Movement artifacts during recordings were detected automatically and validated further by manual inspection of recordings to ensure that only artifact-free episodes were used for analysis. Spectral analysis (i.e. analysis of signal power at individual frequencies expressed as square of the fast Fourier transform (FFT) magnitude) of interictal sequences was performed using the integrated FFT spectral analysis function of NeuroScore. Gamma (30–100 Hz) and theta (4–12 Hz) waveform spectral contributions were quantified by area under the curve (AUC) analysis in 4 one-hour-length sequences (pre-filtered to remove artifact and hypersynchronous spike sequences) per recording. Cross-frequency coupling of theta phase and gamma amplitude was conducted using MATLAB (Mathworks) as previously described [14, 40]. Phase-amplitude distributions and modulation indexes were determined from 8 one-minute-length sequences (artifact- and hypersynchronous spike-free).

### Western blotting

Western blotting was performed as previously described [19]. Primary antibodies were to LAMP5 (Thermo Fisher) and GAPDH (Sigma). Primary antibodies were detected with species specific HRP-coupled secondary antibodies and visualised with the HRP substrate (Bio-Rad) on a ChemiDoc imager (BioRad).

### Statistical analysis

Statistical analysis was done using Graphpad Prism Version 9.2.0. Student’s *t*-tests were performed for pairwise comparison, one-way analysis of variance (ANOVA) tests was used to compare more than two datasets and two-way ANOVA tests were applied to compare groups across time. Linear regression and correlation analysis were conducted by sum-of-squares minimisation. Survival data were analysed by log-rank Mantel–Cox testing. *P* values < 0.05 were considered significant. All values are presented as mean ± standard error of the mean (SEM). Details on individual test parameters and n numbers are provided in figure legends.

## Results

### LAMP5 limits neuronal hyperexcitation in vivo

We have previously shown that induced neuronal hyperexcitation is a reliable proxy for identifying molecular processes involved in tau-dependent network aberrations in AD models [1, 15, 18]. Here, we combined our model of induced brain hyperexcitation [15, 18] with the Collaborative Cross (CC) forward genetics mouse platform [6]. The CC is a large collection of ~ 500 fully genotyped inbred strains originating from intercrossing of 8 founder strains A/J (AJ), C57BL/6J (B6), 129S1/SvImJ (129S1), NOD/ShiLtJ (NOD), NZO/HlLtJ (NZO), CAST/EiK (CAST), PWK/PhJ (PWK) and WSB/EiJ (WSB) (Supplementary Fig. 2, online resource). Single nucleotide polymorphism (SNP) annotation established the relative contribution of each founder to the genome of individual CC strains, allowing rapid identification of phenotype-modifying genes through comparison of effect size and genome makeup of multiple CC lines [26]. Accordingly, we induced excitotoxic seizures with pentylenetetrazol (PTZ; 50 mg/kg i.p.) in at least 10 mice of each of 59 CC strains and the 4 available founder strains, resulting in different severity and latency of progression of seizures between strains (Fig. 1a). Using mean seizure severity for quantitative trait locus (QTL), mapping identified a 135 Mbp susceptibility locus on chromosome 2 with major WSB allele and minor AJ and CAST contributions, while the B6 allele conferred protection (Fig. 1b; Supplementary Table 3; Supplementary Fig. 3, online resource). After examination of coding variants from all 8 founder strains and exclusion of splice sites, only 2 genes remained in the locus of interest; (i) *Lamp5* with a 9-nucleotide insertion (dbSNP: rs224715897) resulting in early truncation at aa 22, a likely a loss of function, and (ii) *Plcb1* (dbSNP: rs258044618) resulting in a missense mutation that was well-tolerated according to the SIFT algorithm [28]. Neuronal *Lamp5* was largely uncharacterised but more recently emerged as a marker of inhibitory interneurons [13]. Using CRISPR/Cas9 genome editing [49], we deleted the transcriptional start in exon 1 of the *Lamp5* locus in C57Bl/6 mice (Fig. 1c; Supplementary Fig. 1, online resource), resulting in loss of LAMP5 protein expression in homozygous *Lamp5*^Δ/Δ^ brains (Fig. 1d). Induction of excitotoxic seizure confirmed a gene-dosage dependent increase in severity and reduced seizure latency in *Lamp5*^Δ/+^ and *Lamp5*^Δ/Δ^ mice, respectively, as compared to *Lamp5*^+/+^ wild-type littermate controls (Fig. 1e), consistent with a novel seizure-limiting role of LAMP5.

### Loss of LAMP5 interneurons in AD brains and models

**Fig. 2:**
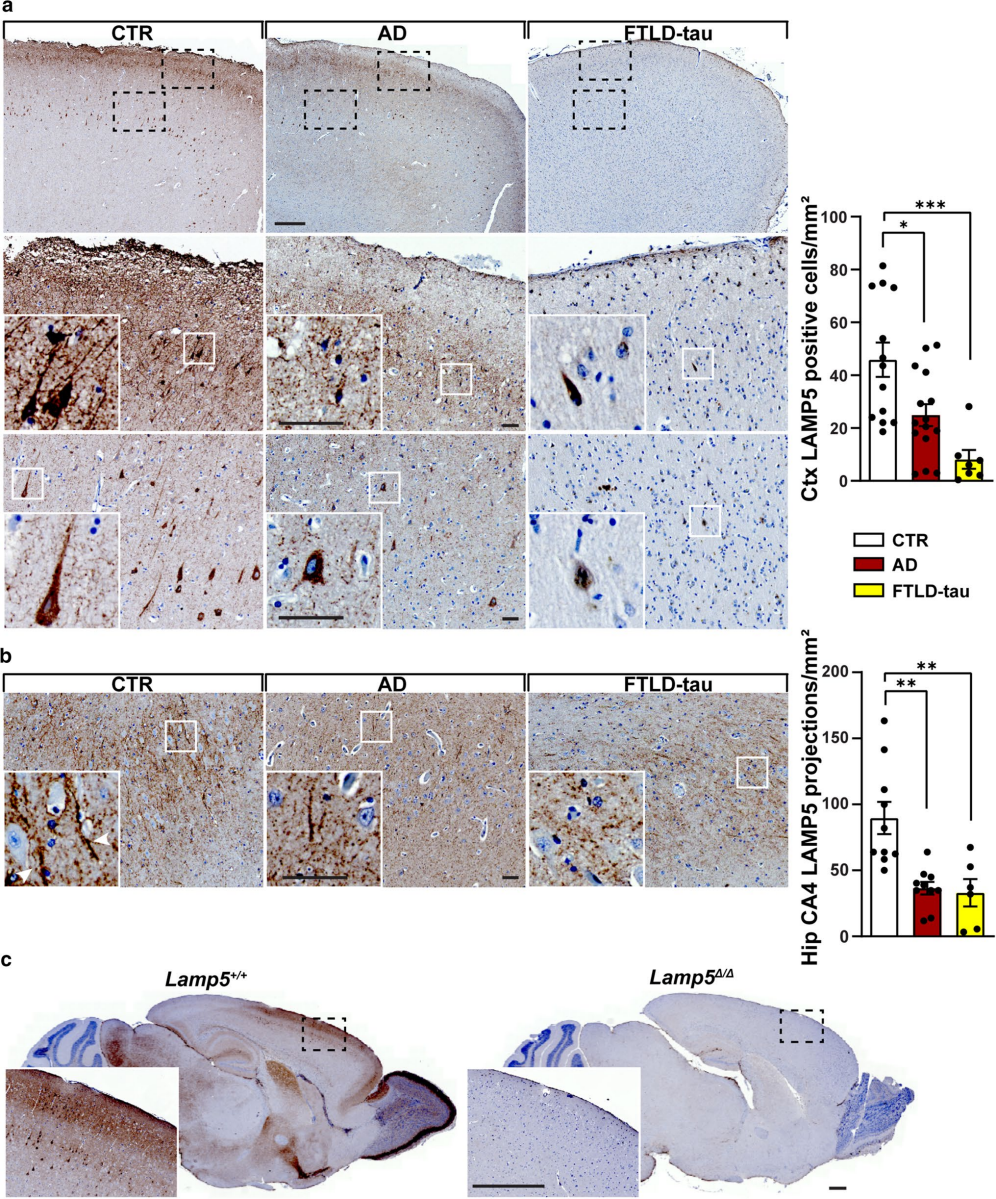
Loss of LAMP5 interneurons in human AD and FTLD. **a** Representative staining of LAMP5 (brown; arrowheads in CTR) in the frontal cortex from human AD, FTLD-tau and control (CTR) brains. Overview (top row) and higher magnification (broken boxes) of layer I/II (middle row) and layer IV (bottom row) are shown. Insets show individually stained neurons. Quantification of numbers of LAMP5+ neurons (*p < 0.05; ***p < 0.001; Student *t* test). Scale bar, top row 500 μm, middle and bottom row 50 μm. **b** Representative staining of LAMP5 (brown; arrowheads in CTR) in the hippocampus (CA4) from human AD, FTLD-tau and control (CTR) brains. Quantification of LAMP5+ projections (**p < 0.01; Student *t* test). Scale bar, 50 μm. **c** Sagittal sections of wild-type (*Lamp5*^+/+^) and LAMP5 deficient (*Lamp5*^Δ/Δ^) mouse brains stained for LAMP5 (brown) confirms antibody specificity. Higher magnification show frontal cortex (broken box). Scale bar, 500 μm

**Fig. 3:**
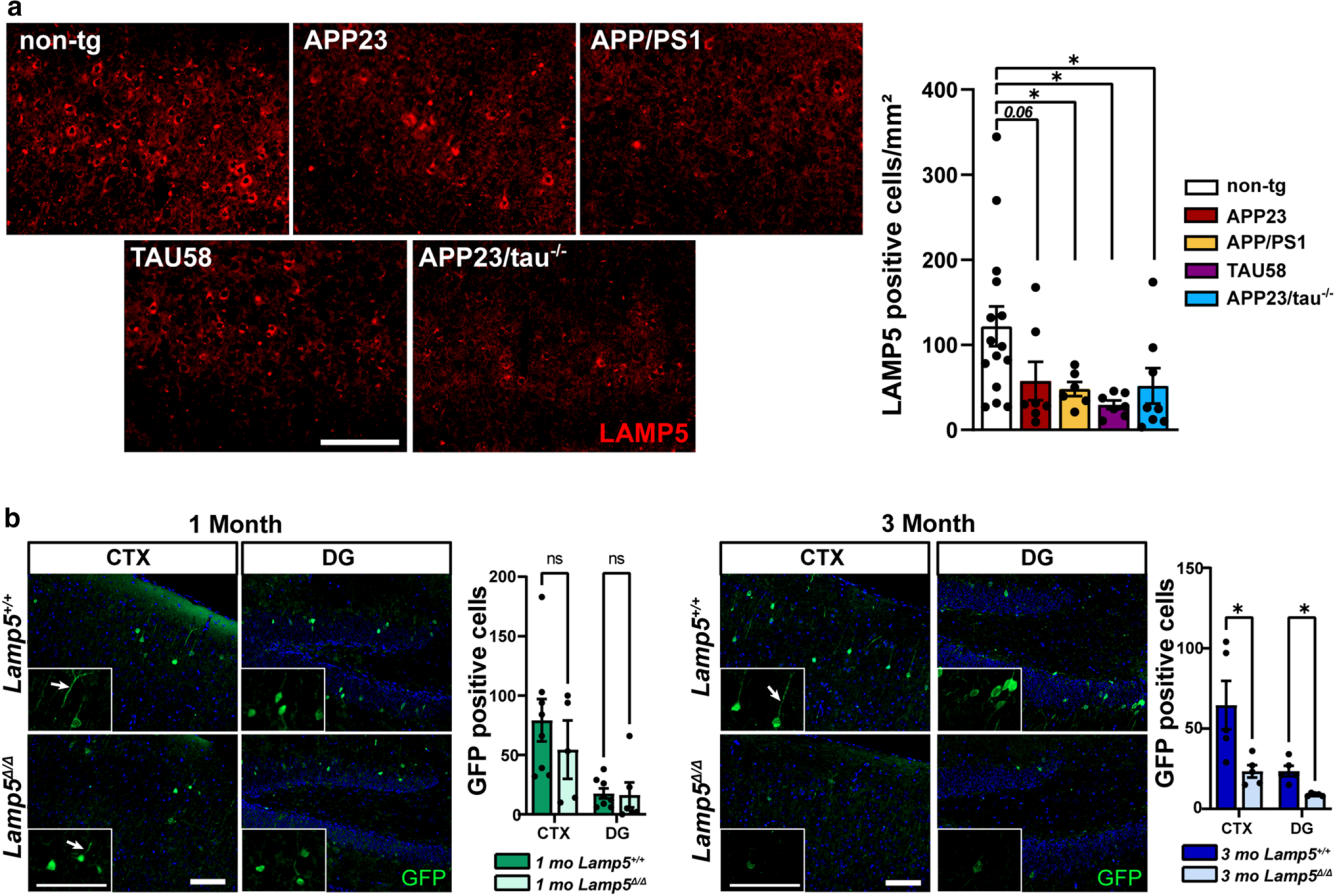
Loss of LAMP5 interneurons in Aβ and tau-expressing mouse models of AD and FTLD-tau. **a** Representative staining of LAMP5 in 12-month-old of control (non-tg), APP23, APP/PS1, TAU58 and APP23/tau^–/–^ brains. Quantification of numbers of LAMP5+ interneurons in the cortex of non-tg, APP23, APP/PS1, TAU58 and APP23/tau^–/–^ mice (*p < 0.05, Student *t* test). Scale bar, 100 μm. **b** Representative imaging of AAV-mediated eGFP reporter activity controlled by the murine Lamp5 promoter in wild-type (Lamp5^+/+^) and Lamp5^Δ/Δ^ mice at 1 and 3 months of age. Insets show higher magnification of eGFP reporter-positive neurons, including lack of dendritic arborization in 3-month-old Lamp5^Δ/Δ^ brains. Quantification of cells with Lamp5 promoter-driven eGFP reporter expression in the cortex (CTX) and dentate gyrus (DG) of Lamp5^+/+^ and Lamp5^Δ/Δ^ mice (*p < 0.05; Student *t* test). Scale bar, 100 μm

Given our previous experience with translatability of molecular pathways that modulated induced seizures to pathomechanism in AD models [15, 18], we hypothesized that LAMP5 may similarly modulate neuronal hyperexcitation in AD pathogenesis. Recent single-cell sorting studies of mouse brains have identified a non-overlapping subpopulation of inhibitory interneurons expressing LAMP5 but its function(s) is yet-to-be-defined [11, 13, 37]. Cortical LAMP5-positive (LAMP5+) interneurons vary in abundance and distribution between species with mice having less and primates/humans having more [22]. Therefore, we stained frontal cortex and hippocampus sections of human AD, tau-only Frontotemporal lobar degeneration (FTLD-tau), as well as neurologically healthy controls (CTR) lacking neuropathological signs of neurodegenerative diseases and AD, with LAMP5-specific antibodies (Fig. 2; Supplementary Table 2; Supplementary Fig. 4 and 5, online resource). Specificity of antibody staining of neurons, their projections and synaptic terminals was confirmed using *Lamp5*^Δ/Δ^ brain sections (Fig. 2c). All AD and FTLD-tau cases presented with a marked reduction of LAMP5+ neurons and their projections/synaptic boutons in the frontal cortex as compared to CTR (Fig. 2a). Consistently, LAMP5 containing neuronal projections were significantly reduced in the hippocampus of AD and FTLD-tau cases compared to CTR (Fig. 2b). Similarly, globus pallidus, where LAMP5 is highly expressed in neuronal projections, showed significant LAMP5 reduction in AD (Supplementary Fig. 6, online resource). To understand whether this is a result of the underlying Aβ and/or tau pathology, we determined LAMP5 expression in brains of APP23 mice with transgenic neuronal expression of human mutant APP [36] and APP/PS1 mice co-expressing human mutant APP and presenilin 1 (PS) [2], both forming Aβ, as well as TAU58 mice that express neuronal human mutant tau and form neurofibrillary pathology [44]. Similar to human AD and FTLD-tau, we found an age-dependent significant loss of LAMP5+ neurons in brain sections of APP23, APP/PS1 and TAU58 mice as compared to non-transgenic (non-tg) littermates (Fig. 3a). Interestingly, aged tau-deficient APP23/tau^−/−^ brains showed comparable loss of LAMP5+ neurons as do APP23 mice, suggesting the Aβ induced loss of LAMP5 expression in AD is not tau-dependent. Notably, we previously reported only partially improved survival and recovery of behavioural deficits in APP23/tau^−/−^ mice compared to APP23 alone [15, 18]. LAMP5 loss may explain this incomplete rescue of APP23/tau^−/−^. It was critical to establish whether reduced LAMP5 signals were due to reduced protein expression or actual loss of LAMP5+ interneuron populations. Therefore, we injected *Lamp5*^Δ/Δ^ mice and *Lamp5*^+/+^ littermates with adeno-associated viruses (AAVs) encoding a *Lamp5* promoter-driven green fluorescence protein (*pLamp5*-GFP) reporter. This labelled a distinct neuronal population in *Lamp5*^+/+^ controls consistent with spatial and temporal distribution of cortical and hippocampal LAMP5+ interneurons (Fig. 3b, Supplementary Fig. 7, online resource). At 1 months of age, reporter signals were comparable in *Lamp5*^+*/*+^ and *Lamp5*^Δ/Δ^ cortex and hippocampus, suggesting normal development of LAMP5+ interneurons in these mice. In contrast, 3 months-old *Lamp5*^Δ/Δ^ mice presented with marked reduction of *pLamp5*-GFP reporter-expressing neurons and dystrophic neurites consistent with loss of LAMP5+ interneurons due to LAMP5 reduction. This suggests that a reduction and loss of LAMP5 causes degeneration of LAMP5+ interneurons in cortex and dentate gyrus and hence, loss of their inhibitory functions.

### LAMP5 reduction accelerates deficits of APP23 mice

**Fig. 4:**
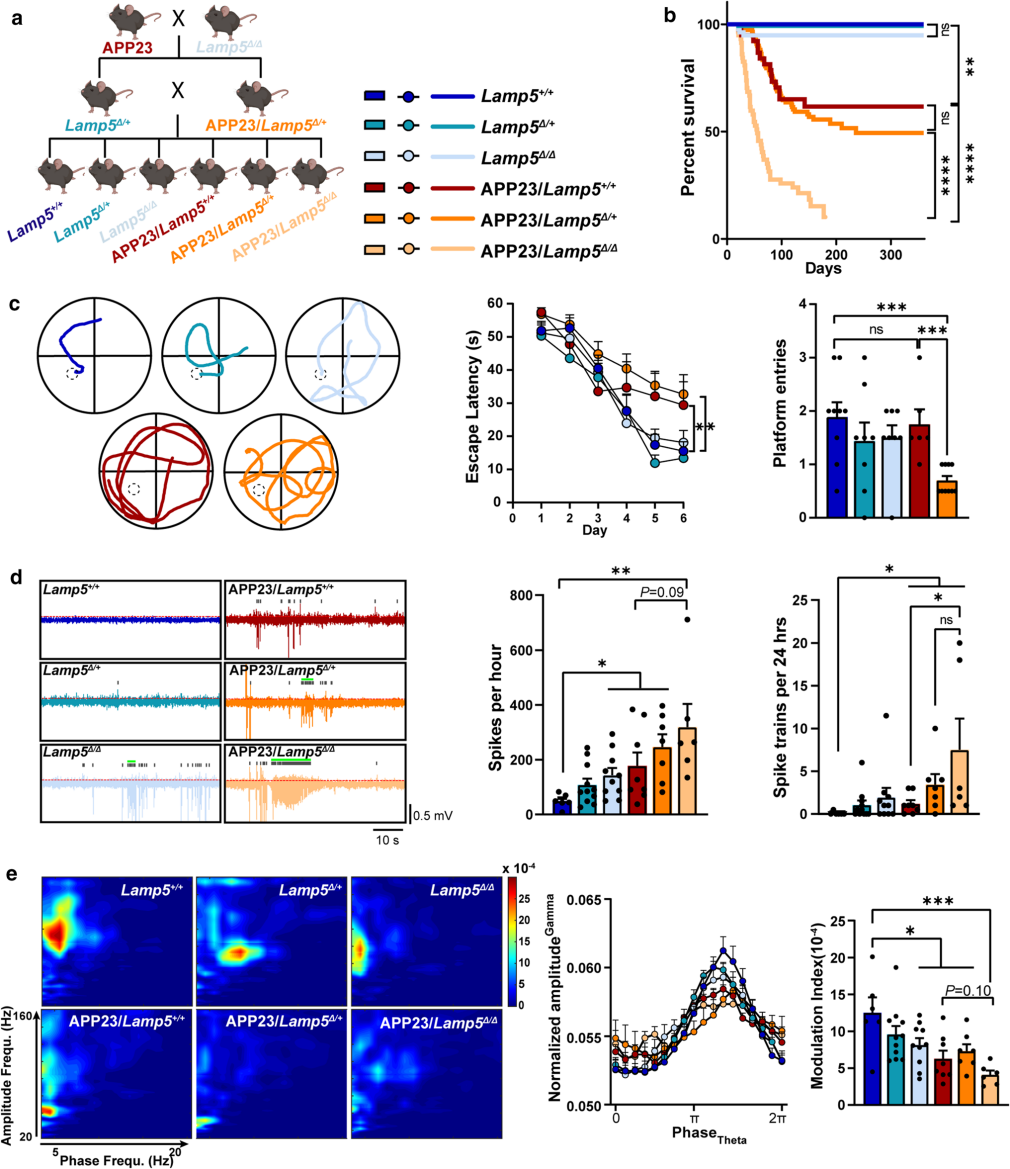
LAMP5 reduction augments deficits in AD mice. **a** Crossbreeding scheme of APP23 and Lamp5^Δ/Δ^ strains to obtain experimental colony. Color-coding of indicated genotypes apply to all figure panels. **b** Survival of Lamp5^+/+^ (*n* = 39), Lamp5^Δ/+^ (*n* = 158), Lamp5^Δ/Δ^ (*n* = 90), APP23/Lamp5^+/+^ (*n* = 40), APP23/Lamp5^Δ/+^ (*n* = 118), APP23/Lamp5^Δ/Δ^ (n = 59) mice (**p < 0.01; ****p < 0.0001; ns, not significant; Mantel–Cox test). **c** Memory testing at 12 months of age in the Morris water maze: example shown of day 6 swim traces per genotype (left), mean latency to find escape platform on individual days of the acquisition trials (middle) and platform zone entries during probe trials without escape platform on day 7 (right) (*p < 0.05; ***p < 0.001; ns, not significant; two-way ANOVA (Tukey post hoc) for acquisition trials; one-way ANOVA for probe trials). **d** Hippocampal electroencephalography (EEG) recording in freely moving mice at 4.5 months of age: example of EEG traces showing episodes of spontaneous hyperexcitatory discharges (spikes: grey bars; spike trains: green bars) in *Lamp5*^Δ/Δ^ and APP23 crossings (*left*), quantification of spontaneous hyperexcitatory discharges (= spikes) per hour (*middle*) and occurrence of artifact-corrected spike trains during 24 h EEG recordings (*right*) (*p < 0.05; **p < 0.01; ns, not significant; one-way ANOVA (Tukey post hoc)). **e** Interictal cross-frequency coupling (CFC) of theta phase and gamma amplitude was disrupted in *Lamp5*^Δ/Δ^ and APP23 crossings (*left*). Phase amplitude blots of interictal EEG recording with reduced CFC in APP23/*Lamp5*^Δ/+^ and more so in APP23/*Lamp5*^Δ/Δ^ mice (*middle*). Modulation index of CFC (*right*) (*p < 0.05; ***p < 0.001; ns, not significant; one-way ANOVA (Tukey post hoc))

To directly address whether LAMP5 reduction is an effect of Aβ neuropathology or contribute to neuronal network aberrations in AD, we crossed APP23 mice onto a *Lamp5*^Δ/Δ^ background to deplete LAMP5 expression (Fig. 4a). APP23, like other APP transgenic lines, are characterized by a significant premature mortality that has been attributed to early onset neuronal hyperexcitation [15, 18, 32]. Accordingly, male and more so, female APP23/*Lamp5*^+/+^ mice showed a significantly reduced survival as compared to non-tg littermates (Fig. 4b, Supplementary Fig. 8, online resource). Survival of APP23/*Lamp5*^Δ/+^ mice was comparable to APP23/*Lamp5*^+/+^ littermates until 150 days and thereafter showed a trend towards increased mortality. In contrast, APP23/*Lamp5*^Δ/Δ^ mice presented with earlier and more rapid mortality and poor overall survival, suggesting a gene dosage effect. Survival of *Lamp5*^Δ/Δ^ and *Lamp5*^Δ/+^ mice was not compromised. Aβ formation and plaque load in aged APP23/*Lamp5*^Δ/+^ mice were comparable to APP23/*Lamp5*^+/+^ littermates (Supplementary Fig. 9, online resource). Learning and memory formation was impaired in APP23/*Lamp5*^Δ/+^ compared to APP23/*Lamp5*^+/+^ mice at 12 months of age (Fig. 4c, Supplementary Fig. 10, online resource). Memory testing of APP23/*Lamp5*^Δ/Δ^ mice was prohibited by their high premature mortality rate prior to deficits become overt in APP23 mice [43]. Before memory deficits manifest, 2-month old APP23 mice presented with hyperactivity and slower habituation in the open field task as compared to non-tg littermates (Supplementary Fig. 10, online resource). This was augmented in APP23/*Lamp5*^Δ/Δ^ mice with significantly increased hyperactivity over the entire test period. *Lamp5*^Δ/Δ^ and *Lamp5*^Δ/+^ did not affect overall activity but delayed habituation as compared to non-tg mice. Consistent with our previous findings [14], hippocampal electroencephalography (EEG) recording showed significantly increased spontaneous discharges and spike trains in APP23/*Lamp5*^+/+^ mice as compared to non-tg controls (Fig. 4d). Spectral power analysis showed previously reported increased high frequency power (β < γ) in APP23/*Lamp5*^+/+^ mice compared to non-tg controls, which was similarly increased in *Lamp5*^Δ/Δ^ mice (Supplementary Fig. 11, online resource). Interestingly, LAMP5 depletion alone was sufficient to produce frequent spontaneous discharge events and increased high frequency power in the absence of Aβ (Fig. 4d, Supplementary Fig. 11, online resource). Both spontaneous discharges and spike trains were further increased in APP23/*Lamp5*^Δ/Δ^ mice. In addition, spectral power at gamma (30–100 Hz) frequency was markedly increased in APP23/*Lamp5*^Δ/+^ and more so in APP23/*Lamp5*^Δ/Δ^ mice as compared to APP23/*Lamp5*^+/+^ littermates, revealing profound neuronal network hypersynchronicity upon LAMP5 reduction in APP23 mice. Accordingly, θ/γ cross frequency coupling (CFC), an EEG measure linked to memory in humans [4, 5, 10, 41] which is compromised in APP23 [14] mice, was further disrupted in APP23/*Lamp5*^Δ/+^ and APP23/*Lamp5*^Δ/Δ^ mice compared to APP23/*Lamp5*^+/+^ littermates (Fig. 4e). Taken together, LAMP5 reduction accelerated and worsened mortality and functional deficits associated with Aβ in AD mice.

### Lamp5 reduction augments impairments in TAU58 mice

**Fig. 5:**
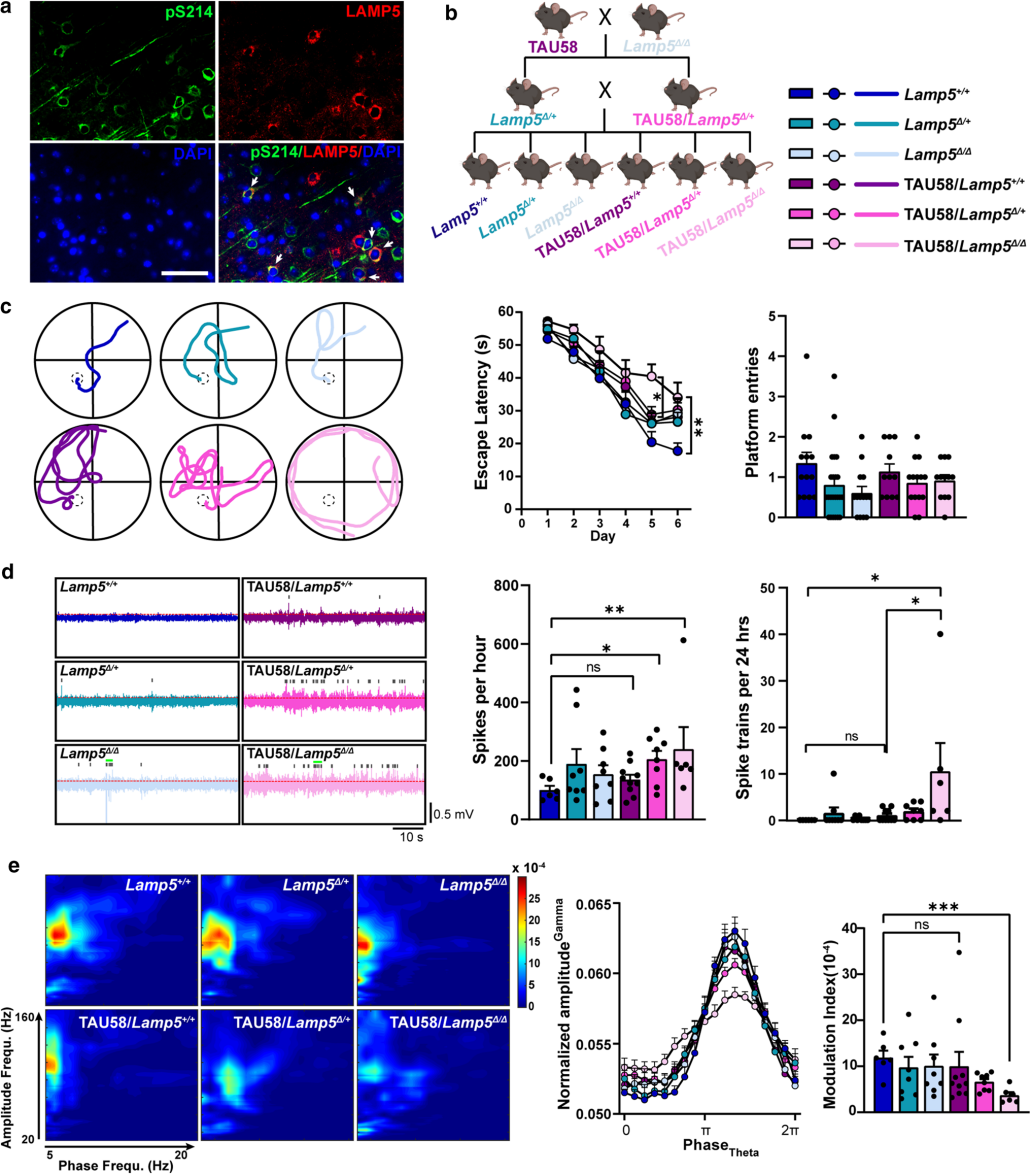
LAMP5 aggravates impairments of P301S tau transgenic mice. **a** Representative staining of pS214 (green) and LAMP5 (red) in 3-month-old TAU58 brains. Arrows indicate cells with pS214 and LAMP5 colocalization. Scale bar, 50 μm. **b** Crossbreeding scheme of TAU58 and Lamp5^Δ/Δ^ strains to obtain experimental colony. Color-coding of indicated genotypes apply to all figure panels. **c** Memory testing at 6 months of age in the Morris water maze: example shown of day 6 swim traces per genotype (left), mean latency to find escape platform on individual days of the acquisition trials (middle) and platform zone entries during probe trials without escape platform on day 7 (right) (*p < 0.05; **p < 0.01; two-way ANOVA (Tukey post hoc) for acquisition trials; one-way ANOVA for probe trials). **d** Hippocampal electroencephalography (EEG) recording in freely moving mice at 6.5 months of age: example shown of EEG traces showing episodes of spontaneous hyperexcitatory discharges (spikes: grey bars; spike trains: green bars) in *Lamp5*^Δ/Δ^ and TAU58 crossings, and increase amplitudes in TAU58/*Lamp5*^Δ/+^ and TAU58/*Lamp5*^Δ/Δ^ mice (*left*), quantification of spontaneous hyperexcitatory discharges (= spikes) per hour (*middle*) and occurrence of artifact-corrected spike trains during 24 h EEG recordings (*right*) (*p < 0.05; **p < 0.01; ns, not significant; one-way ANOVA (Tukey post hoc)). (**e**) Interictal cross-frequency coupling (CFC) of theta phase and gamma amplitude was disrupted in *Lamp5*^Δ/Δ^, TAU58/Lamp5^Δ/+^ and more so in TAU58/Lamp5^Δ/Δ^ mice (left). Phase amplitude blots of interictal EEG recording with reduced CFC in TAU58/*Lamp5*^Δ/+^ and more so in TAU58/*Lamp5*^Δ/Δ^ mice (middle). Modulation index of CFC (*right*) (***p < 0.001; ns, not significant; one-way ANOVA (Tukey post hoc))

Having identified the loss of LAMP5+ interneurons in human FTLD-tau brains and tau transgenic mice (Figs. 2a, 3a), we further assessed if tau pathology develops in LAMP5+ interneurons per se. Consistent with a recent study showing accumulation of phosphorylated tau in GABAergic interneurons in human AD and AD mice brains [50], phosphorylated tau was detected in LAMP5+ interneurons in the cortex of 3-months old TAU58 mice (Fig. 5a). Considering other neuronal populations harbouring tau pathology are not lost in TAU58 mice, LAMP5+ interneurons may be more vulnerable to toxic tau species. To determine the functional consequences of LAMP5 depletion on tau-induced deficits and pathology in the absence of Aβ pathology, we crossed the *Lamp5*^Δ/Δ^ line with TAU58 mice which express P301S human tau in neurons (Fig. 5b) [44]. TAU58 mice develop tau pathology with progressive functional deficits, including impaired learning and neuronal network dysfunction [30]. LAMP5 depletion did not change tau pathology in TAU58/*Lamp5*^Δ/Δ^, TAU58/*Lamp5*^Δ/+^ and TAU58/*Lamp5*^+/+^ mice (Supplementary Fig. 12, online resource), nor was their overall survival compromised (Supplementary Fig. 13a, online resource). However, learning of TAU58/*Lamp5*^Δ/Δ^ mice was significantly impaired compared to TAU58/*Lamp5*^Δ/+^ and TAU58/*Lamp5*^+/+^ mice and non-tau transgenic controls (Fig. 5c, Supplementary Fig. 13, online resource). Consistent with learning deficits, EEG recording of TAU58/*Lamp5*^Δ/+^ and more so TAU58/*Lamp5*^Δ/Δ^ mice revealed increased numbers of spontaneous discharges (Fig. 5d). Furthermore, high frequency spectral power (β/γ) was significantly elevated, while low frequency spectral power (θ) was reduced in TAU58/*Lamp5*^Δ/Δ^ compared to TAU58/*Lamp5*^+/+^ littermates (Supplementary Fig. 14, online resource). Accordingly, CFC was severely compromised in TAU58/*Lamp5* ^Δ/Δ^ mice (Fig. 5e). Hence, loss of LAMP5 augmented the impact of tau pathology on neuronal network function in FTLD-mutant tau transgenic mice, consistent with the loss of inhibitory interneuron function.

## Discussion

In summary, we identified *Lamp5* as a novel modifier gene that limits neuronal hyperexcitation and contributes to physiological brain network functions. This is in line with LAMP5 expression in a small subpopulation of hippocampal and cortical inhibitory interneurons [13, 22]. Aβ/Tau pathology-dependent loss of LAMP5+ interneurons in AD mouse models and human AD/FTLD-tau may therefore directly contribute to the neuronal network aberrations and symptoms reported in the disease models and affected humans. While previous LAMP5 studies mainly focus on its role in cancer [8, 35, 47], insights into its function in the brain remain limited, despite its brain-specific expression pattern [21]. Interestingly, *Lamp5* knockout mice displayed increased amplitudes of auditory brainstem responses [21], indicative of enhanced neuronal activity in the cochlear system. *Lamp5* knockout also has decreased striatal GABAergic neurotransmitter release probability [38]. Although the *C. elegans* LAMP5 homolog UNC-46 localized to presynaptic terminals of inhibitory GABAergic interneurons [33] similar to LAMP5 in mice [21, 38] and humans (this study), its role of pre-synaptic VGAT delivery was not conserved from *C. elegans* to mice [21, 38]. No neuronal loss was reported in basal ganglia and brainstem of *Lamp5* knockout mice [21, 38], but these studies focused on very young mice in the absence of additional stress, like Aβ and tau in the present study, or investigated the less abundant cortical and hippocampal LAMP5+ neurons. The present study is the first report of a functional role of specific LAMP5+ inhibitory interneurons in disease. Although rare in numbers, the inhibitory function of hippocampal and/or cortical LAMP5+ interneurons appear to be critical for limiting hyperexcitation of executive neuron populations susceptible to Aβ/tau-induced impairment in the pathogenesis of AD and FTLD-tau. This explains, at least in parts, the neuronal network aberration observed in human AD and AD mice. Mechanistically, we found that both Aβ and tau induced loss of LAMP5+ interneurons, engaging either converging or parallel pathways. Whether these specific interneurons or LAMP5 function(s) represent potential therapeutic targets for AD and FTLD-tau remains to be shown.

## Supplementary Information

Below is the link to the electronic supplementary material.

Supplementary file1 (PDF 14140 kb)

## Data Availability

Plasmids and sequences of the *pLamp5*-GFP reporter are available upon request and will be deposited to Addgene. All data that support the findings in this study are available from the corresponding author upon request.
