# Supplementary material for: Loss of LAMP5 interneurons drives neuronal network dysfunction in Alzheimer’s disease
Source: Acta Neuropathol. 2022 Jul 3;144(4):637–50. doi: 10.1007/s00401-022-02457-w (PMC9467963; doi:10.1007/s00401-022-02457-w)
Supplement: Supplementary file 1 — Supplementary file1 (PDF 14140 kb) [file 401_2022_2457_MOESM1_ESM.pdf]

Supplementary Data for

Loss of LAMP5 interneurons drives neuronal network dysfunction in Alzheimer's disease

Yuanyuan Deng, Mian Bi, Fabien Delerue, Shelley L. Forrest, Gabriella Chan, Julia van der Hoven, Annika van Hummel, Astrid F. Feiten, Seojin Lee, Ivan Martinez-Valbuena, Tim Karl, Gabor G. Kovacs, Grant Morahan, Yazi D. Ke, Lars M. Ittner

*Correspondence to lars.ittner@mq.edu.au

Content

Supplementary Tables 1 to 3

Supplementary Figures 1 to 14

Supplementary Tables

Table S1. Oligonucleotide primers used for genotyping of mouse strains

Strain	Forward primer (5’-3’)	Reverse primer (5’-3’)
APP23	GTTCTGCTGCATCTTGGACA	GAATTCCGACATGACTCAGG
TAU58	AAGTCACCCAGCAGGGAGGTG	TGTCTCCAATGCCTGCTTCTTC
$tau^{-/-}$	AAGTTCATCTGCACCACCG	TGCTCAGGTAGTGGTTGTCG
$Lamp5^{\Delta/\Delta}$	GCAGATACCATTGCAGAGCA	TGTGGCTCTCTGGGAGTTCT
APPswe	AGGACTGACCACTCGACCAG	CGGGGGTCTAGTTCTGCAT
PSEN1	AATAGAGAACGGCAGGAGCA	GCCATGAGGGCACTAATCAT

Table S2. Clinical and neuropathological details of human samples

Group	Age (y)	Sex	PMI	Thal phase	CERAD score	Braak stage	TDP-43	α-synuclein	Region
Ctr	62	M	19	0	-	0	no	no	CTX, PU
Ctr	44	M	14	0	-	0	no	no	CTX, HIP, GP, PU
Ctr	61	M	20.5	0	-	0	no	no	CTX, HIP, GP, PU
Ctr	50	M	36	0	-	0	no	no	CTX, HIP, GP, PU
Ctr	56	M	14	0	-	0	no	no	CTX, HIP, GP, PU
Ctr	80	F	36	2	-	2	no	no	CTX, HIP
Ctr	83	M	10.5	1	-	1--2	no	no	CTX
Ctr	73	M	8	1	-	1	no	no	CTX
Ctr	75	F	17	2	-	2	no	no	CTX
Ctr	65	M	29	0	-	2	no	no	CTX
Ctr	70	F	13	0	-	1	no	no	CTX
Ctr	68	F	23	0	-	1	no	no	CTX
Ctr	66	M	8	0	-	2	no	no	CTX, HIP
**Ctr**	**65.6 ± 3.1**	**M**	**19.1 ± 2.7**	**0.46 ± 0.2**		**0.91 ± 0.3**	**no**	**no**	
Ctr	77	M	15	-	1	0	no	no	HIP
Ctr	79	M	8	0	0	0	no	no	HIP
Ctr	64	F	5	1	0	0	no	no	HIP
Ctr	68	M	11	0	0	0	no	no	HIP
**Ctr**	**72.0 ± 3.6**		**9.8 ± 2.1**		**0.3 ± 0.3**	**0**	**no**	**no**	**HIP**
AD	78	M	21	5	-	6	no	no	CTX, HIP, GP, PU
AD	64	M	16	5	-	6	no	no	CTX, GP, PU
AD	80	F	2	5	-	5	no	no	CTX, HIP, GP, PU
AD	56	F	1	5	-	5	no	no	CTX, HIP, GP, PU
AD	79	F	18	4	-	5	no	no	CTX, HIP, GP, PU
AD	80	F	2	5	-	6	no	no	CTX, HIP
AD	79	M	4	5	-	6	no	no	CTX, HIP
AD	77	F	10	5	-	6	no	no	CTX, HIP
AD	62	M	-	5	-	6	1	Amy	CTX
AD	66	F	-	5	-	6	no	no	CTX
AD	78	M	-	5	-	6	no	no	CTX
AD	60	M	-	5	-	6	no	limbic	CTX
AD	64	M	-	5	-	6	1	limbic	CTX
AD	62	F	-	5	-	6	1	limbic	CTX
AD	59	M	-	5	-	6	no	Amy	CTX
**AD**	**69.6 ± 2.4**			**4.9 ± 0.1**		**5.8 ± 0.1**	**-**	**-**	
AD	78	M	13	-	3	6	no	no	HIP
AD	68	M	23	3	3	5	no	no	HIP
AD	70	M	6	-	3	6	no	no	HIP
**AD**	**72.0 ± 3.1**		**14.0 ± 4.9**		**3**	**5.7 ± 0.3**	**no**	**no**	**HIP**
FTLD-tau	68	M	6.5	-	-	-	no	no	CTX
FTLD-tau	73	F	7.5	-	-	-	no	no	CTX
FTLD-tau	89	F	11	-	-	-	no	no	CTX
FTLD-tau	75	M	9	-	-	-	no	no	CTX
FTLD-tau	87	F	36	-	-	-	no	no	CTX
FTLD-tau	71	M	23	-	-	-	no	no	CTX
FTLD-tau	67	F	18	-	-	-	no	no	CTX
FTLD-tau	**75.7 ± 3.3**		**15.9 ± 4.1**				**no**	**no**	**CTX**
FTLD-tau	71	F	6	-	0	0	no	no	HIP
FTLD-tau	65	F	3	-	0	4	no	no	HIP
FTLD-tau	76	F	9	-	0	0	no	no	HIP
FTLD-tau	62	F	9	-	0	0	no	no	HIP
FTLD-tau	78	M	26	-	0	0	no	no	HIP
FTLD-tau	76	F	9	-	0	0	no	no	HIP
**FTLD-tau**	**71.3 ± 2.7**		**10.3 ± 3.3**		**0**	**0.7± 0.7**	**no**	**no**	**HIP**

PMI, post-mortem interval; CTX, cortex; PU, Putamen; GP, Globus pallidus; HIP, hippocampus; Amy, amygdala ‘-‘, data not available; mean ± SEM of group in bold.

Table S3. Odds ratio of founder strains conferring susceptibility on Chr 2

Strain	Susceptible	Non Susceptible	Total Alleles	Odds Ratio	p-value (FDR)
AJ	6	2	8	5.00	ns
BL6	2	26	28	0.08	***
129S1	2	10	12	0.27	ns
NOD	8	14	22	0.83	ns
NZO	9	16	25	0.81	ns
CAST	5	0	5	infinite	ns
PWK	6	4	10	2.43	ns
WSB	10	0	10	infinite	***

***, p<0.001; ns, not significant

Supplementary Figures

Supplementary Figure 1

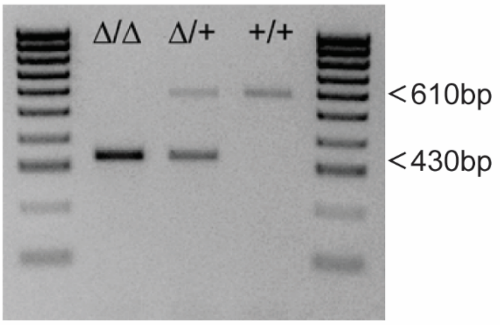

***Lamp5*$^{\Delta/\Delta}$ strain genotyping.** Representative genotyping PCR demonstrating 250bp deletions in mice derived from M1 founder. Homozygous deletion of Lamp5 (Δ/Δ), heterozygous deletion (Δ/+) and wild-type (+/+) mice are shown here.

Supplementary Figure 2

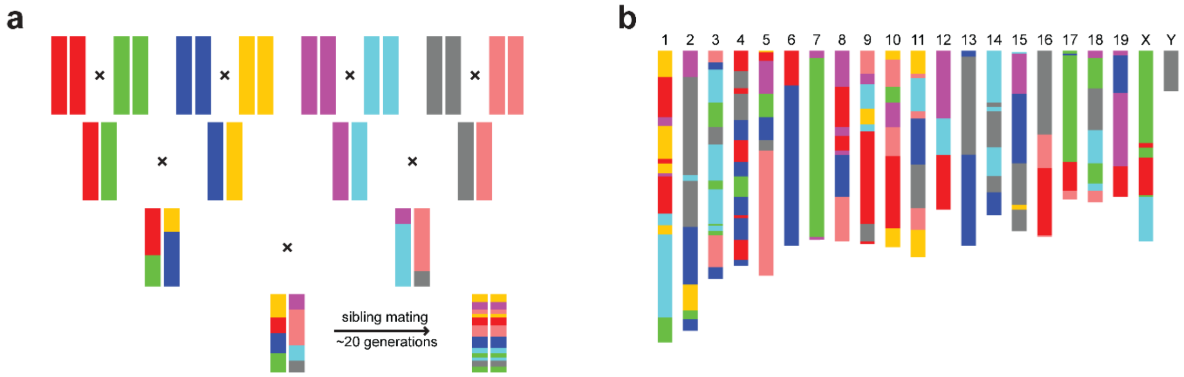

The Collaborative Cross. (**a**) To establish the Collaborative Cross (CC) platform, 8 founder strains (different colour chromosomes) are crossed in random permutation to derive four G1 hemizygous strains. These are crossed to yield a G3 population where the inbreeding process begins for another 20 generations. (**b**) An exemplified chromosome arrangement from a CC strain. All murine chromosomes with colour denoting parental origin of genomic segments from (a). After 20 generations of inbreeding, the estimated residual hemizygosity is less than 10%.

Supplementary Figure 3

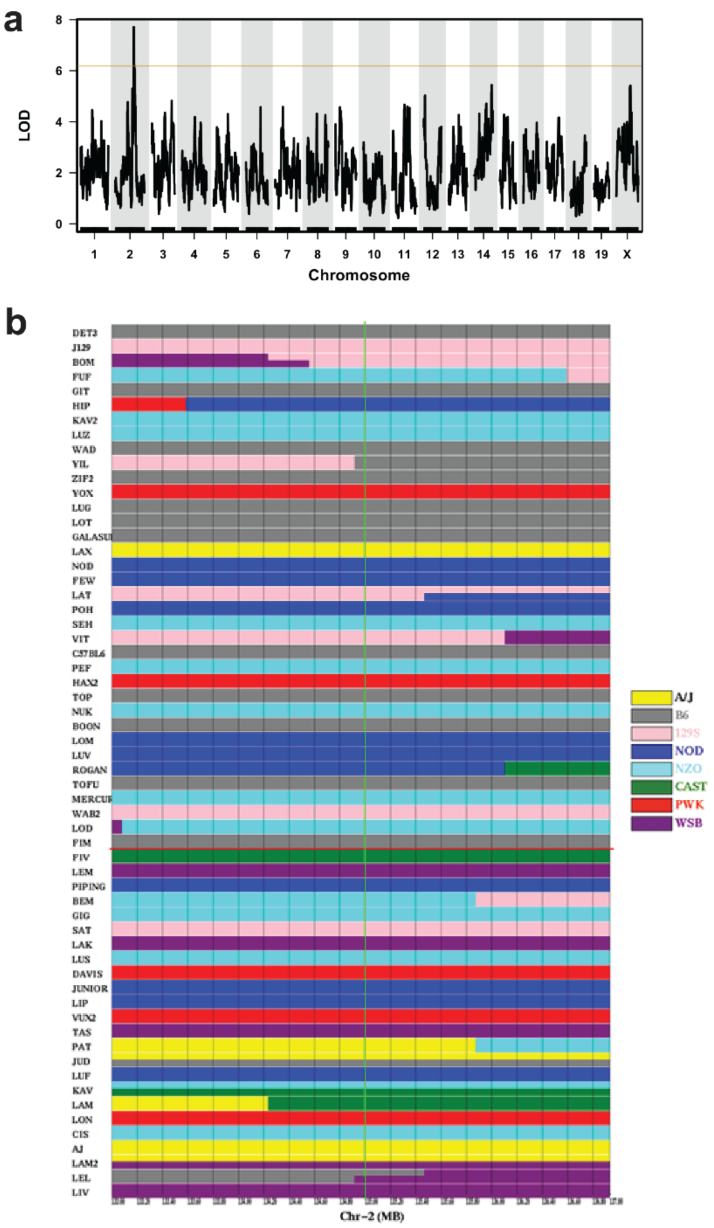

QLT mapping. (**a**) Manhattan blot of QTL mapping using Mean Seizure Severity showing a locus on chromosome 2. (**b**) Haplotype of each strain contributing to the phenotype is shown. Haplotype calls are made based on contribution at 135.0 MB of the murine chromosome 2 (green line) given the frequency of recombination within this region. Animals with mean Seizure Severity Score of less than 3 are shown above the red line and greater or equals to 3 below the red line.

Supplementary Figure 4

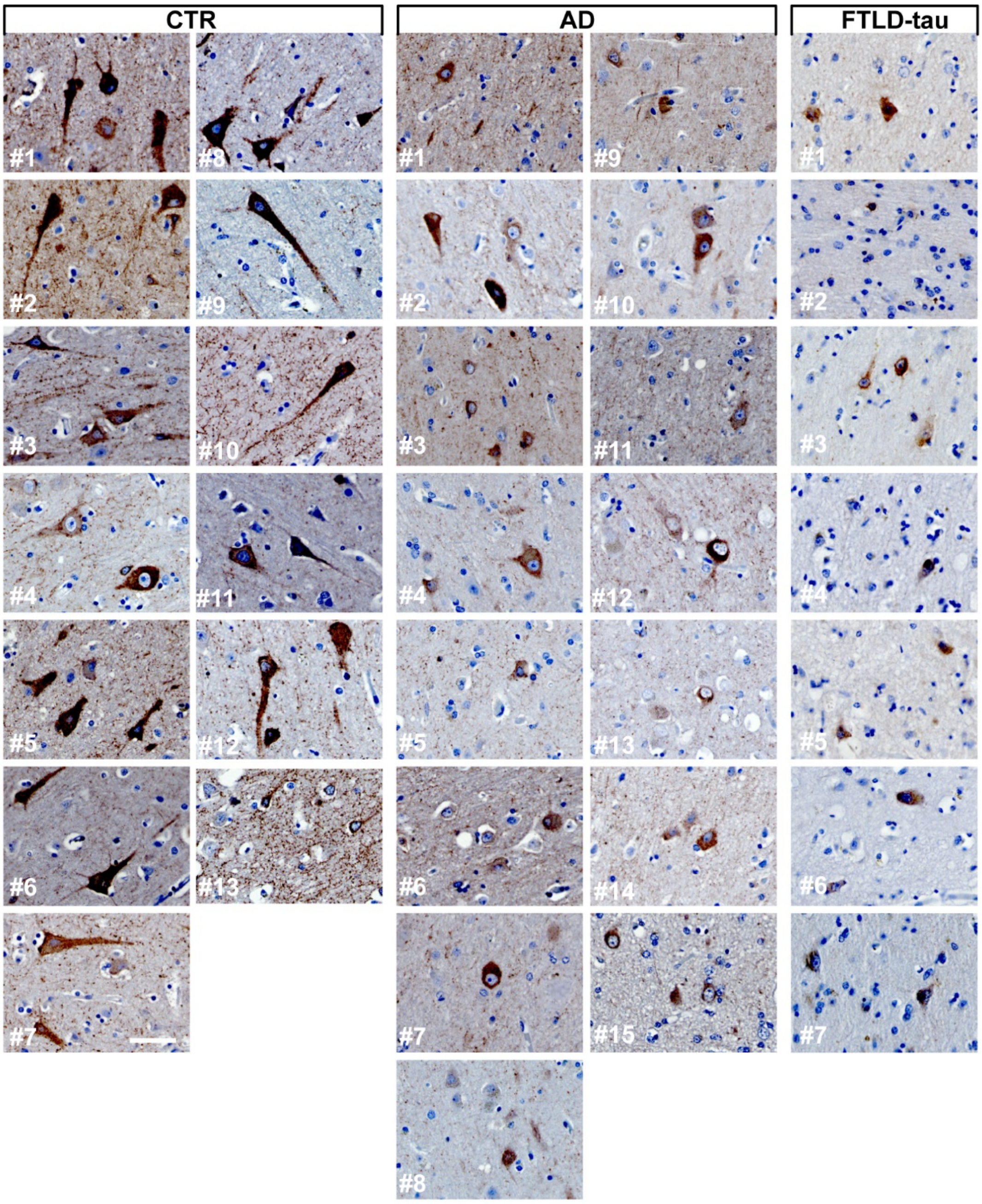

LAMP5 staining in the cortex of Alzheimer's disease, FTLD-tau and control brains. Representative staining of LAMP5 (brown) in the cortex from all human AD, FTLD-tau and control (CTR) brains. Scale bar, 50 µm.

Supplementary Figure 5

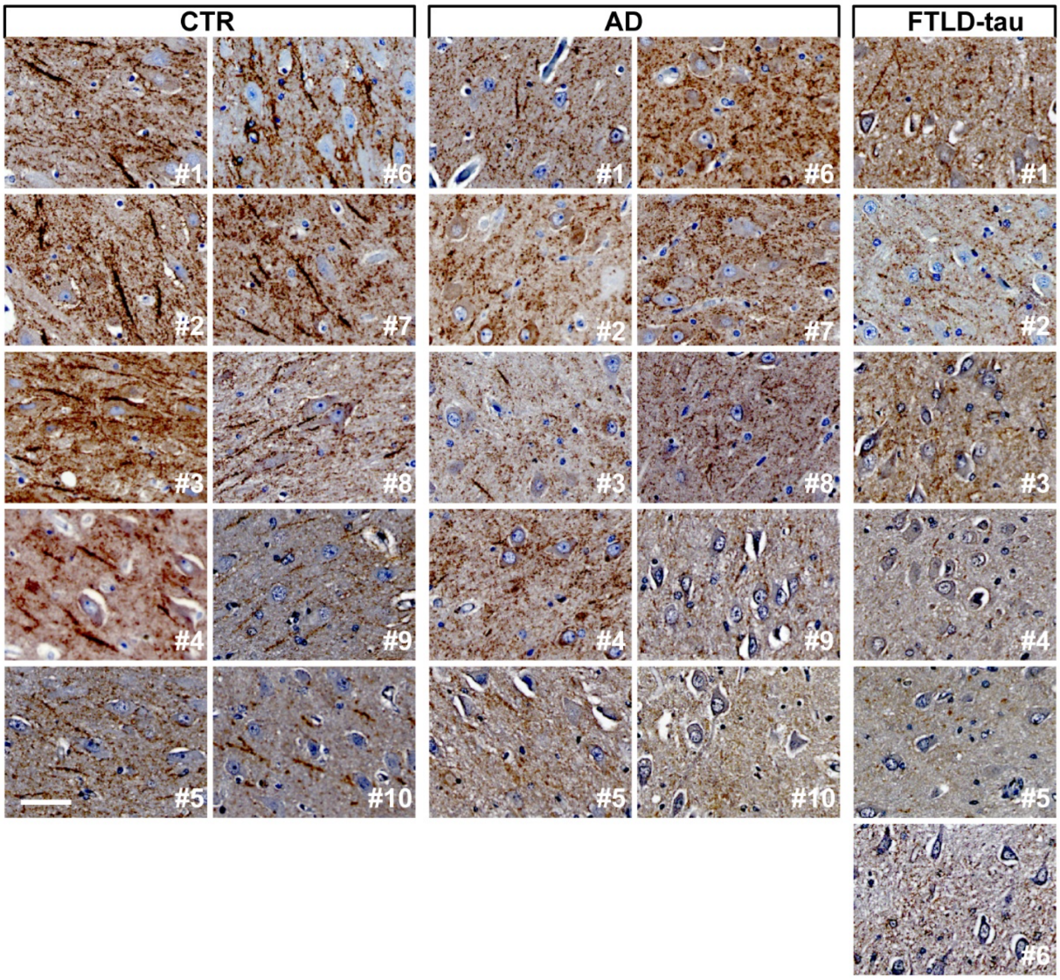

LAMP5 staining in the hippocampus of Alzheimer's disease, FTLD-tau and control brains. Representative staining of LAMP5 (brown) in the hippocampus (CA4) from all human AD, FTLD-tau and control (CTR) brains. Scale bar, 50 µm.

Supplementary Figure 6

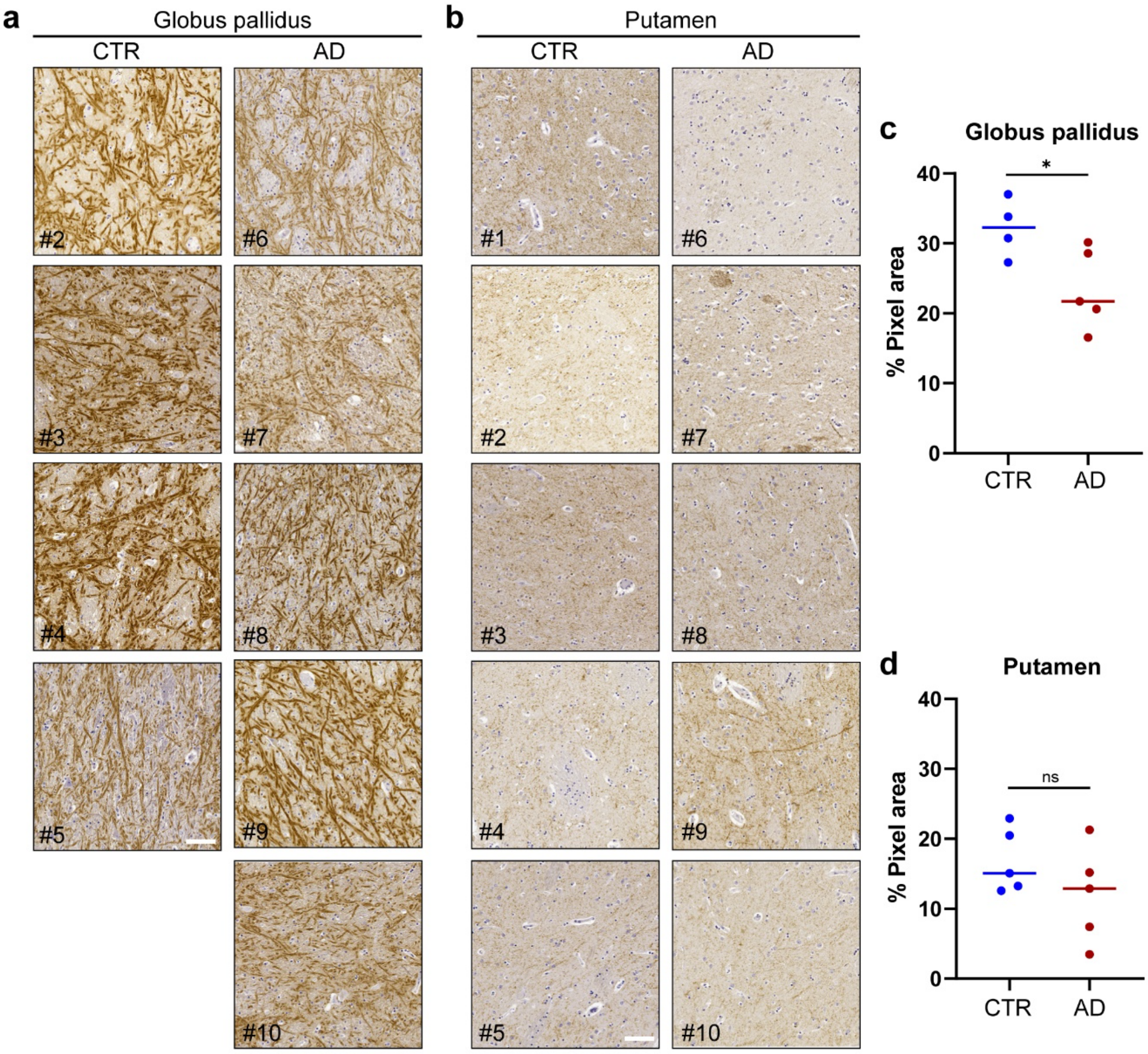

LAMP5 staining in the basal ganglia of Alzheimer's disease and control brains. (**a**) Representative staining of LAMP5 (brown) in the globus pallidus from human AD and control (CTR) brains. No globus pallidus tissue was available for case #1. Scale bar, 100 µm. (**b**) Representative staining of LAMP5 (brown) in the putamen from human AD and control (CTR) brains. Scale bar, 100 µm. (**c**) Quantification of LAMP5+ staining in the globus pallidum (*, $p<0.05$; Student *t* test). (**d**) Quantification of LAMP5+ staining in the putamen (ns, not significant; Student *t* test).

Supplementary Figure Supplementary Figure 7

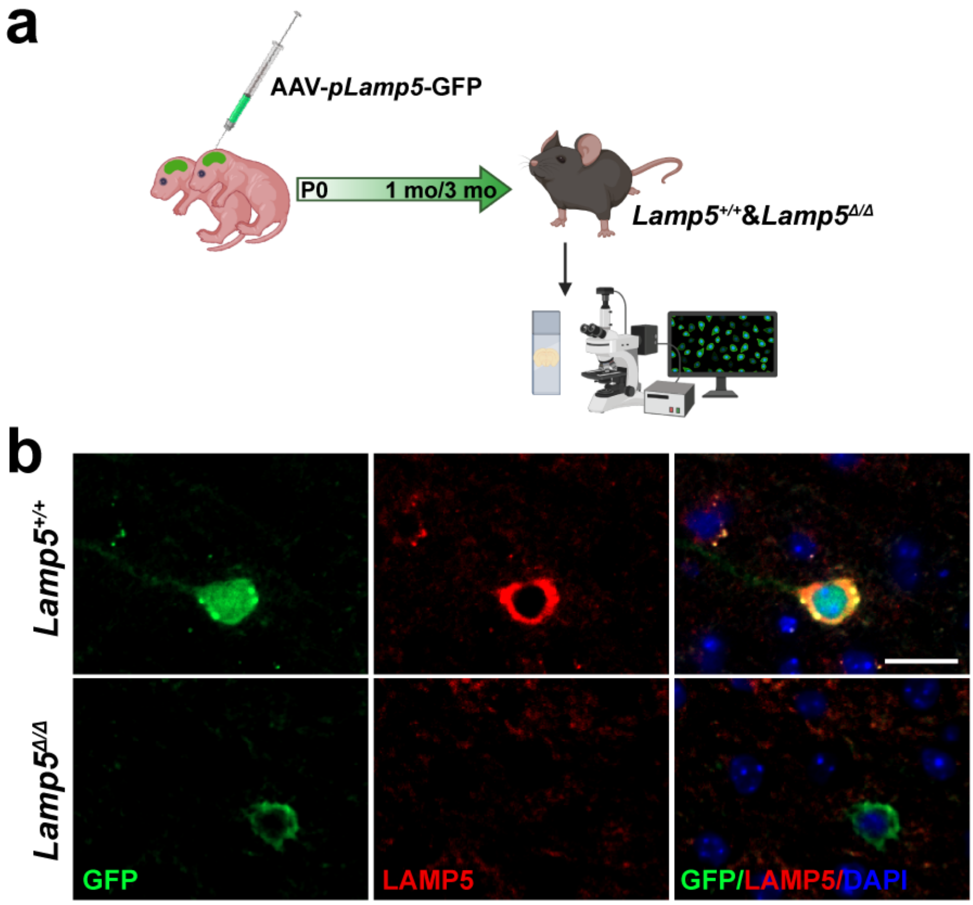

Murine *Lamp5* reporter. (**a**) experimental design for *Lamp5* reporter expression experiments. Newborn wild-type *Lamp5*$^{+/+}$ and *Lamp5*$^{\Delta/\Delta}$ littermates were injected with adeno-associated virus (AAV) for expression of green fluorescence protein (GFP) under control of the murine *Lamp5* promoter. At 1 and 3 months of age, GFP expression was imaged. (**b**) Staining of AAV-*pLamp5*-GFP brains at 1 months of age with antibodies to LAMP5 conformed expression of GFP (green) in LAMP5+ (red) cells in the brains of wild-type *Lamp5*$^{+/+}$ mice. No GFP expression was observed in LAMP5-negative cells in the brains of wild-type *Lamp5*$^{+/+}$ mice. Nuclei were visualized with DAPI (blue). Scale bar, 20 µm.

Supplementary Figure 8

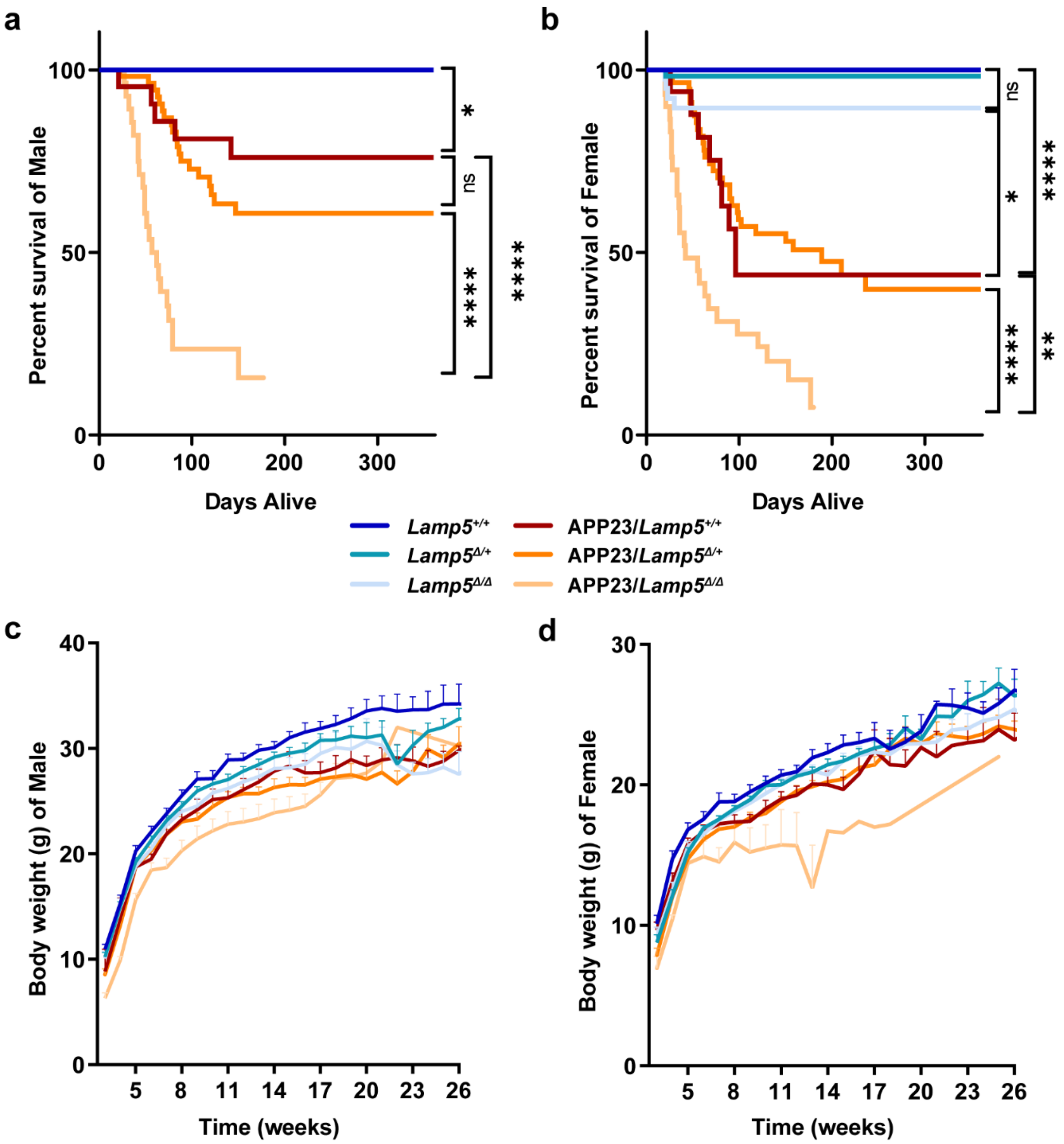

Sex-specific survival and body weights of APP23/*Lamp5*$^{\Delta/\Delta}$ mice. (**a**) Survival of male Lamp5$^{+/+}$ (n=23), Lamp5$^{\Delta/+}$ (n=94), Lamp5$^{\Delta/\Delta}$ (n=56), APP23/Lamp5$^{+/+}$ (n=22), APP23/Lamp5$^{\Delta/+}$ (n=60), APP23/Lamp5$^{\Delta/\Delta}$ (n=29) mice (*, p<0.05; ****, p<0.0001; ns, not significant; Mantel-Cox test). (**b**) Survival of female Lamp5$^{+/+}$ (n=16), Lamp5$^{\Delta/+}$ (n=64), Lamp5$^{\Delta/\Delta}$ (n=44), APP23/Lamp5$^{+/+}$ (n=18), APP23/Lamp5$^{\Delta/+}$ (n=58), APP23/Lamp5$^{\Delta/\Delta}$ (n=30) mice (*, p<0.05; **, p<0.01; ****, p<0.0001; ns, not significant; Mantel-Cox test). (**c-d**) Body weights were not significantly different in male and female mice of the respective genotypes. Body weight tracing was limited by the premature mortality of APP23/*Lamp5*$^{\Delta/\Delta}$.

Supplementary Figure 9

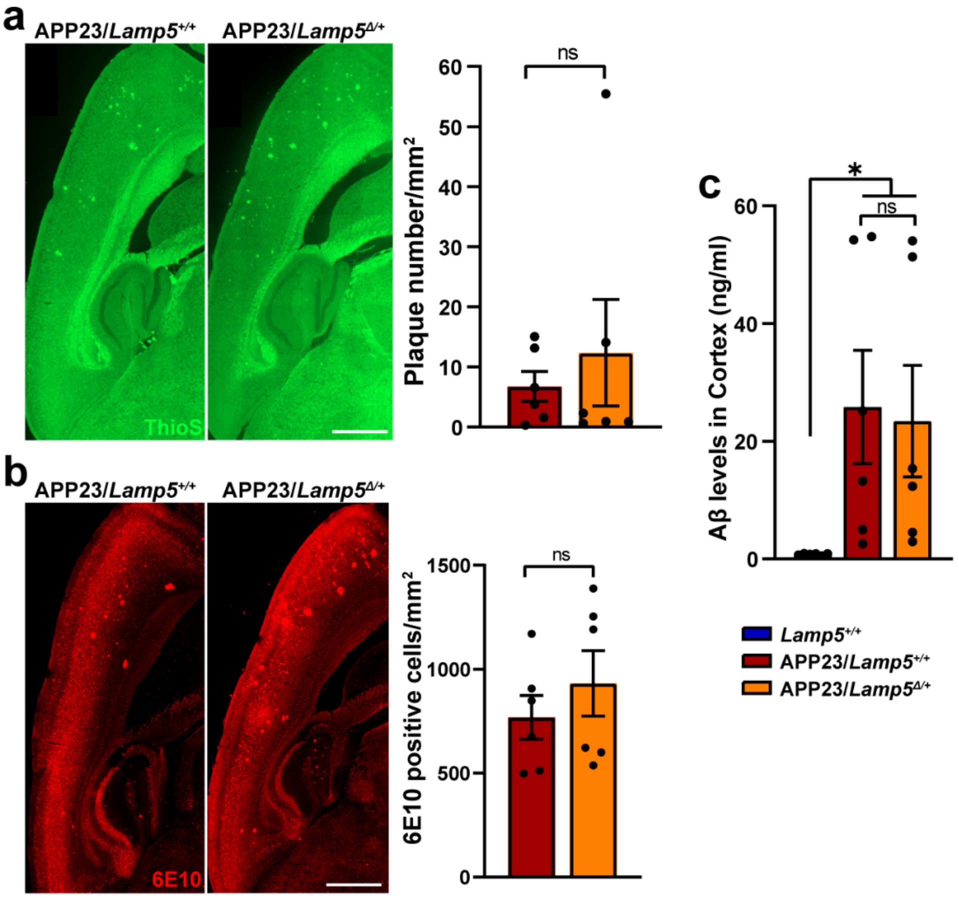

Unchanged Aβ pathology in aged APP23/*Lamp5*$^{\Delta/+}$ mice. (**a**) Representative Thioflavin S (ThioS, green) staining of amyloid-β (Aβ) plaques in 12 months old APP23/*Lamp5*$^{+/+}$ and APP23/*Lamp5*$^{\Delta/+}$ mice. Quantification of ThioS+ plaques (ns, not significant; Student *t* test). Scale bar, 500 µm. (**b**) Representative immunostaining of Aβ (6E10 antibody, red) in 12 months old APP23/*Lamp5*$^{+/+}$ and APP23/*Lamp5*$^{\Delta/+}$ brains. Quantification of 6E10+ cells (ns, not significant; Student *t* test). Scale bar, 500 µm. (**c**) Aβ$_{1\text{-}42}$ levels in the brains of control (*Lamp5*$^{+/+}$) and APP23/*Lamp5*$^{+/+}$ and APP23/*Lamp5*$^{\Delta/+}$ brains (*, $p<0.05$; ns, not significant; repeated Student *t* test).

Supplementary Figure 10

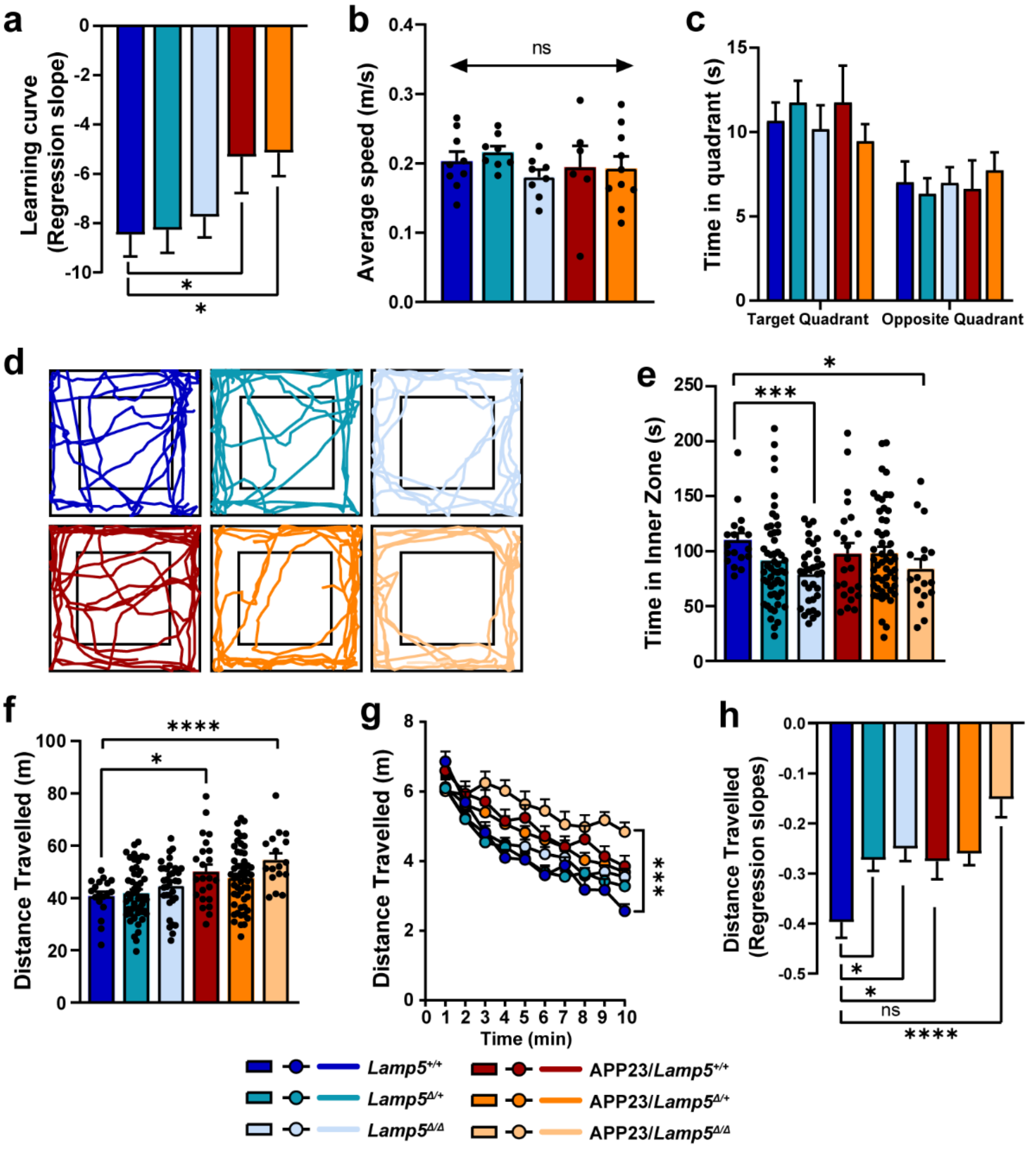

Augmented behavioural deficits in APP23/*Lamp5*$^{\Delta/\Delta}$ mice. (**a**) Linear regression slopes of Morris water maze (MWM) escape latency curves shown in the main Fig. 3c (*, p<0.05; one-way ANOVA). (**b**) Average swim speed during MWM testing (ns, not significant; one-way ANOVA). (**c**) Time spent in target and opposing quadrant during MWM probe trials. (**d-h**) Open field locomotor testing at 2 months of age. (d) example movement traces in the open field arena per genotype. (e) Time spent for inner zone exploration. (f) Total distance travelled in the open field arena. (g) Distance travelled per minute in the open field arena. (h) Linear regression slopes of minute-by-minute distance travelled shown in (h) (*, p<0.05; ***, p<0.001; ****, p<0.0001; ns, not significant; two-way ANOVA (Tukey post hoc) for minute-by-minute analysis; one-way ANOVA for other comparisons).

Supplementary Figure 11

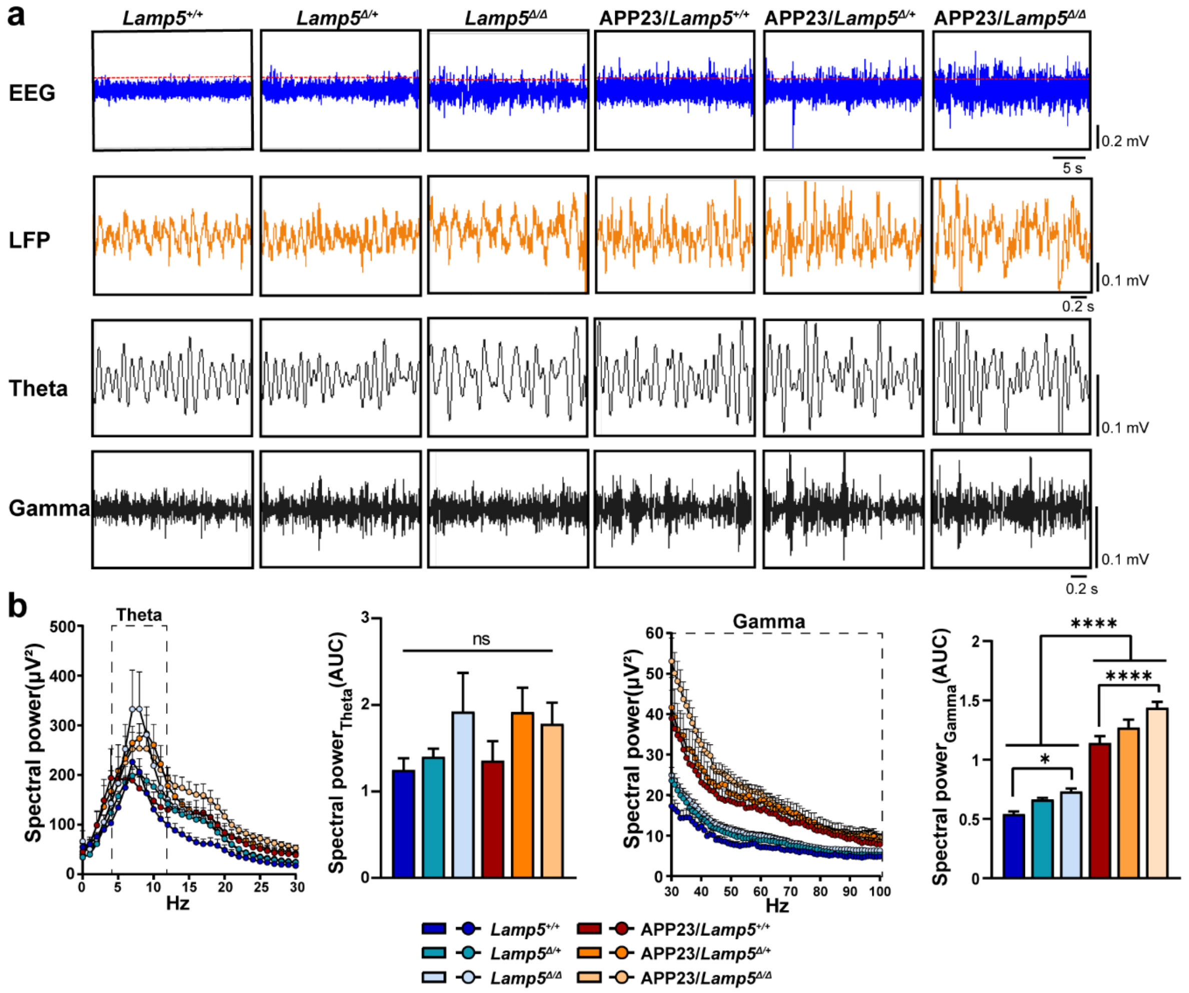

Neuronal network deficits in APP23/*Lamp5*$^{\Delta/\Delta}$ mice. (**a**) Example electroencephalography (EEG) traces, local field potential (LFP) and isolated theta and gamma waves for all indicated genotypes. (**b**) Spectral power of theta (*left*) and gamma (*right*) EEG wave frequencies for indicated genotypes together with area under the curve (AUC) analysis for indicated frequency ranges (broken boxes) (*, $p<0.05$; ****, $p<0.0001$; ns, not significant; one-way ANOVA).

Supplementary Figure 12

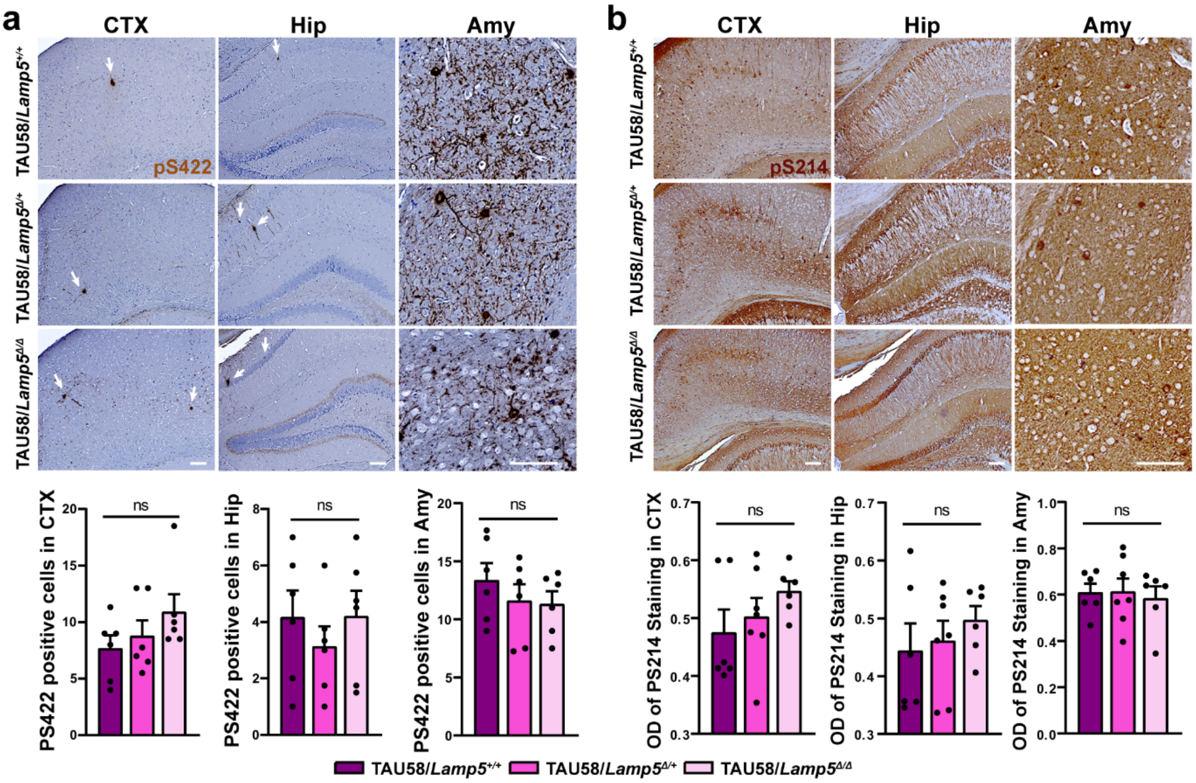

Unchanged tau pathology in mature TAU58/*Lamp5*$^{\Delta/\Delta}$ mice. (**a**) Representative immunohistochemistry with antibodies to tau phosphorylated at Serine 422 (pS422; brown, arrows) in the cortex (CTX) hippocampus (Hip) and amygdala (Amy) of TAU58/*Lamp5*$^{+/+}$, TAU58/*Lamp5*$^{\Delta/+}$ and TAU58/*Lamp5*$^{\Delta/\Delta}$ mice (ns, not significant; one-way ANOVA). Scale bars, 100 µm. (**a**) Representative immunohistochemistry with antibodies to tau phosphorylated at Serine 214 (pS214; brown) in the cortex (CTX) hippocampus (Hip) and amygdala (Amy) of TAU58/*Lamp5*$^{+/+}$, TAU58/*Lamp5*$^{\Delta/+}$ and TAU58/*Lamp5*$^{\Delta/\Delta}$ mice (ns, not significant; one-way ANOVA). Scale bars, 100 µm.

Supplementary Figure 13

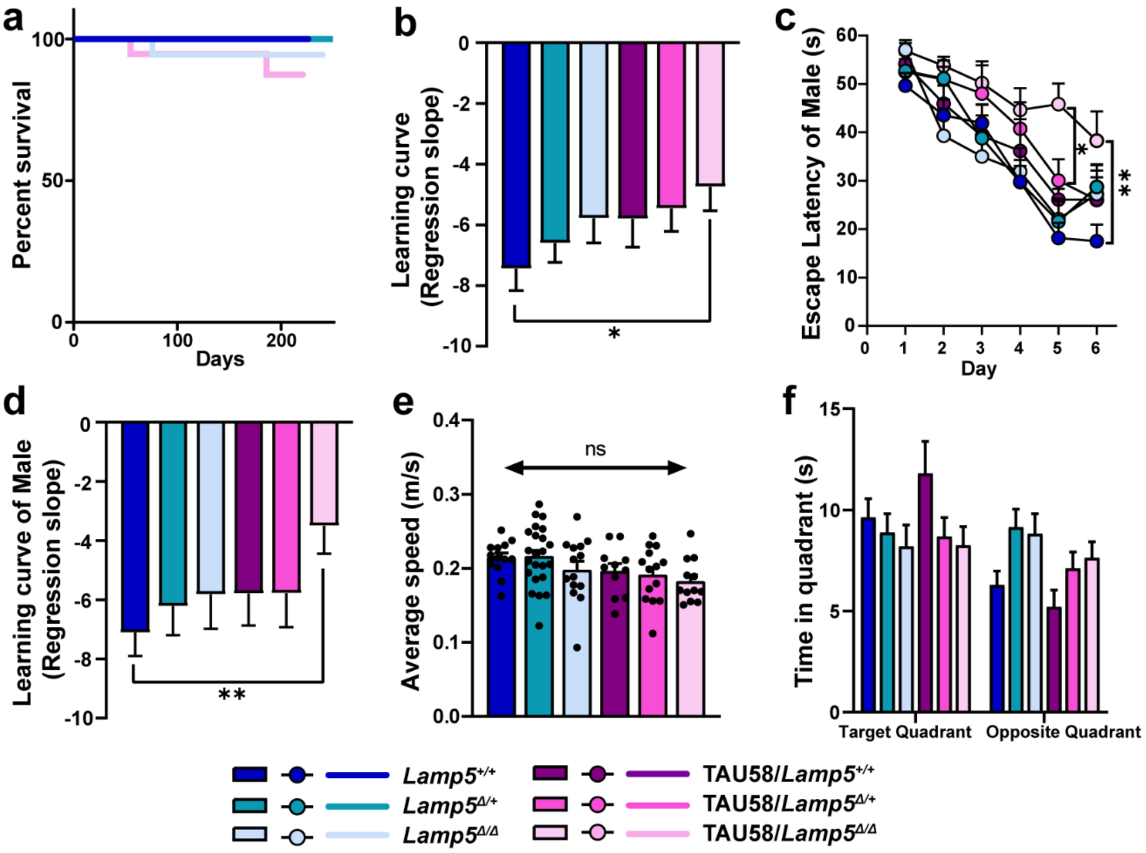

Additional functional data from TAU58/*Lamp5*$^{\Delta/\Delta}$ mice. (**a**) Survival of Lamp5$^{+/+}$ (n=21), Lamp5$^{\Delta/+}$ (n=47), Lamp5$^{\Delta/\Delta}$ (n=22), TAU58/Lamp5$^{+/+}$ (n=17), TAU58/Lamp5$^{\Delta/+}$ (n=34), TAU58/Lamp5$^{\Delta/\Delta}$ (n=21) mice (ns, not significant; Mantel-Cox test). (**b**) Linear regression slopes of Morris water maze (MWM) escape latency curves shown in the main Fig. 4c (*, p<0.05; one-way ANOVA). (**c**) Mean latency for male mice to find escape platform on individual days of the acquisition trials during MWM testing (**, p<0.01; two-way ANOVA (Tukey post hoc)). (**d**) Linear regression slopes of Morris water maze (MWM) escape latency curves shown in (c) (*, p<0.05; one-way ANOVA). (**e**) Average swim speed during MWM testing (ns, not significant; one-way ANOVA). (**f**) Time spent in target and opposing quadrant during MWM probe trials.

Supplementary Figure 14

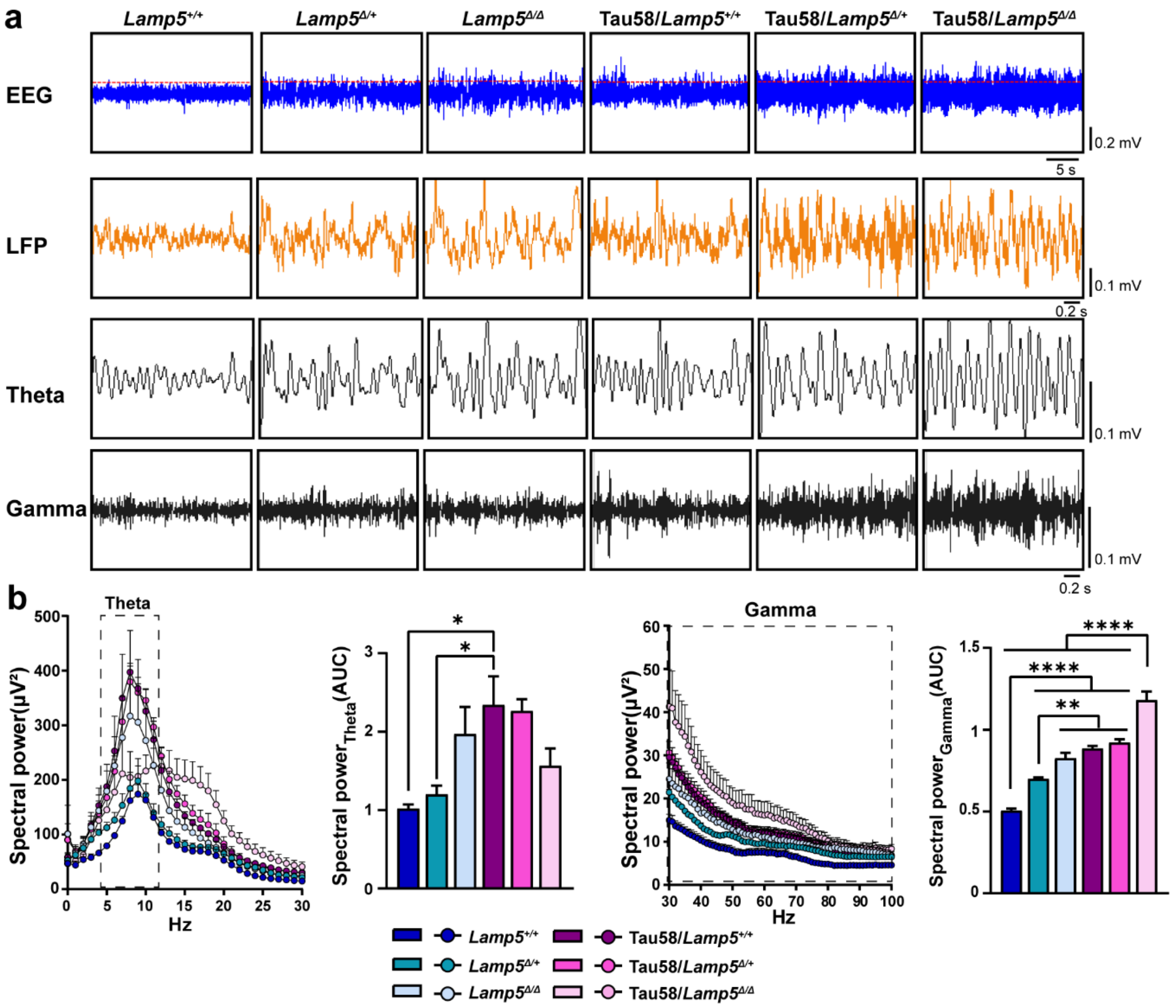

Neuronal network deficits in TAU58/*Lamp5*$^{\Delta/\Delta}$ mice. (**a**) Example electroencephalography (EEG) traces, local field potential (LFP) and isolated theta and gamma waves for all indicated genotypes. (**b**) Spectral power of theta (*left*) and gamma (*right*) EEG wave frequencies for indicated genotypes together with area under the curve (AUC) analysis for indicated frequency ranges (broken boxes) (*, p<0.05; **, p<0.01; ****, p<0.0001; ns, not significant; one-way ANOVA).
